# Homocysteine thiolactone affects paraoxonase 1 activity via altered paraoxonase 1 distribution on high-density lipoprotein particles

**DOI:** 10.1042/BSR20253768

**Published:** 2026-01-22

**Authors:** Rina Kawaguchi, Tsunehiro Miyakoshi, Akira Yoshimoto, Shoichi Hosoya, Yuki Kugii, Takehiko Sasaki, Nobuharu Suzuki, Ryunosuke Ohkawa

**Affiliations:** 1 Clinical Bioanalysis and Molecular Biology, Graduate School of Medical and Dental Sciences, Institute of Science Tokyo, 1-5-45 Yushima, Bunkyo-ku, Tokyo, 113-8510, Japan; 2 Ochanomizu Research Facility, Bioscience Center, Research Infrastructure Management Center, Institute of Science Tokyo, 1-5-45 Yushima, Bunkyo-ku, Tokyo, 113-8510, Japan; 3 Department of Biochemical Pathophysiology, Medical Research Laboratory, Institute for Integrated Research, Institute of Science Tokyo, 1-5-45 Yushima, Bunkyo-ku, Tokyo, 113-8510, Japan

**Keywords:** apolipoprotein A-I, cardiovascular disease, high-density lipoprotein, homocysteine thiolactone, homocysteinylation, paraoxonase 1

## Abstract

Hyperhomocysteinemia is a risk factor of cardiovascular disease (CVD). High-density lipoprotein (HDL) plays an important role in anti-atherosclerosis, with its anti-atherogenic function attributed to HDL-associated proteins such as apolipoprotein A-I (apoA-I) and paraoxonase 1 (PON1). Homocysteine (Hcy) thiolactone modifies lysine residues in proteins, thereby altering their function. Although dysfunction of apoA-I and PON1 has been reported, the precise modification sites and underlying mechanisms have remained unclear. In this study, we aimed to identify Hcy-thiolactone modification sites on apoA-I and PON1. In addition, we sought to clarify the effects of Hcy-thiolactone on PON1 activity and its distribution. Modification sites were analyzed using MALDI-TOF MS. The effects of Hcy-thiolactone on various specimens, including purified proteins, reconstituted HDL (rHDL), HDL collected by ultracentrifugation, and serum samples, were characterized using enzymatic assays measuring three major PON1 activities (arylesterase, paraoxonase, and lactonase) and Western blotting. Our results demonstrated that while some Hcy-thiolactone modification sites were detected on apoA-I, PON1 itself was not directly modified by Hcy-thiolactone. Thiolactonase activity was reduced by Hcy-thiolactone in large HDL particles. Furthermore, a general reduction of PON1 activity and changes in HDL remodeling and distribution were observed in serum samples treated with Hcy-thiolactone. These findings suggest that PON1 dysfunction induced by Hcy-thiolactone is influenced by alterations in HDL remodeling and the enzyme’s distribution on HDL particles. Analysis of PON1’s distribution dynamics under pathological conditions may provide crucial insights into the mechanism of HDL function decline in CVD.

## Introduction

Elevated serum homocysteine (Hcy) levels have been associated with higher cardiovascular disease (CVD) mortality risks [1]. Hcy is an intermediary metabolite in the metabolic pathways of methionine and cysteine. When this pathway is impaired by genetic or nutritional deficiencies, Hcy accumulates in blood and is metabolically converted to Hcy-thiolactone. Hcy-thiolactone is a reactive thioester that modifies lysine residues in proteins. Its toxicity to human endothelium is most likely due to these protein modifications [2,3]. The modification of proteins by Hcy-thiolactone, called *N*-homocysteinylation, damages or alters protein structure and function. For example, *N*-homocysteinylation has been shown to promote the formation of intermolecular aggregation of albumin after oxidation [4], and *N*-homocysteinylated fibrinogen has a higher resistance of clots to lysis [5].

High-density lipoprotein (HDL) is a lipoprotein contributing to protection against CVD through multiple atheroprotective functions, including antioxidant, anti-inflammatory, and reverse cholesterol transport [6]. Apolipoprotein A-I (apoA-I), as the HDL structure protein, plays a crucial role in these functions. However, chemical substances in blood can modify apoA-I, leading to its dysfunction. Previous studies have demonstrated that apoA-I was also modified by Hcy-thiolactone, and this modification has been shown to impair its antioxidant property, characterized by its suppression of diene formation in low-density lipoprotein (LDL) by copper sulfate *in vitro* [7]. In addition, *N*-homocysteinylated apoA-I has been reported to have a lower cholesterol efflux capacity compared with normal apoA-I [8].

Another important functional protein in HDL is paraoxonase 1 (PON1). PON1 is a calcium-dependent enzyme that hydrolyzes lipid hydroperoxides (LOOHs) derived from oxidized LDL [9]. In addition, PON1 inhibits inflammatory responses from macrophages [10] and promotes cholesterol efflux by stimulating expression of cholesterol transporters [11,12]. PON1 also hydrolyzes various substrates, such as aryl esters, organophosphate pesticides, lactones, and others. Therefore, the measurement of PON1 activity using these substrates, such as arylesterase, paraoxonase, and lactonase activity, respectively, is used for CVD evaluation. Some case-control studies have reported that serum PON1 lactonase activity was associated with the development of CVD [13-15]. Another evaluation of PON1 using arylesterase and paraoxonase activities was also useful to predict the CVD risk [16].

Given the established association between hyperhomocysteinemia and CVD, it is plausible that PON1 may also be modified by Hcy-thiolactone, similar to apoA-I. Indeed, one study had reported that purified PON1 treated with Hcy-thiolactone exhibited lower arylesterase and paraoxonase activities. However, despite these observations, the precise mechanism by which Hcy-thiolactone affects PON1 structure on HDL, and whether Hcy-thiolactone binds directly to PON1, has remained unclear.

From a different viewpoint, regarding *in vitro* studies focusing on PON1, typically, either purified PON1 derived from HDL or HDL samples isolated by ultracentrifugation are used as specimens. While these approaches facilitate relatively simple experimental designs, it is important to acknowledge that the ultracentrifugation process can lead to the dissociation of some HDL-associated proteins including PON1. Furthermore, the presence of various plasma proteins in a native serum environment represents additional factors that could indirectly affect HDL proteins and their interactions. These considerations highlight the need for careful analysis of HDL in different experimental contexts.

Therefore, in this study, we first aimed to identify the Hcy-thiolactone modification sites on apoA-I and PON1 using MALDI-TOF MS, as the specific sites of Hcy-thiolactone modification on these crucial HDL-associated proteins remain largely uncharacterized. Subsequently, to clarify the factors regulating PON1’s susceptibility to Hcy-thiolactone treatment and its activity under various conditions, we investigated the effects of Hcy-thiolactone treatment on PON1 in different specimens, including recombinant PON1 (rePON1), reconstituted HDL (rHDL), ultracentrifuged HDL (uHDL), and whole serum samples.

## Materials and methods

### Antibodies

Monoclonal rabbit anti-PON1 antibody and polyclonal goat anti-rabbit IgG conjugated to horseradish peroxidase (HRP) were purchased from Abcam plc. (Cambridge, U.K.). Polyclonal goat anti-apoA-I antibody was purchased from Academy Bio-Medical Company Inc. (TX, U.S.A.), and polyclonal rabbit anti-goat IgG (HRP) was from Medical & Biological Laboratories Co. Ltd. (Tokyo, Japan).

### Blood samples

Blood samples were collected from healthy volunteers, who provided written informed consent at Tokyo Medical and Dental University (currently the Institute of Science Tokyo). To obtain serum samples, whole blood was centrifuged at 2000 ***g*** for 15 min at 4°C after allowing the blood to clot for 15 min. The isolated serum samples were stored at −80°C until use for experiments.

### Preparation of apolipoprotein B-depleted serum

Apolipoprotein B-depleted serum (BDS) was prepared as described previously [17]. Briefly, 100 µl of serum was mixed with 40 µl of 20% polyethylene glycol (PEG; 6000 m.w.) in 200 mM glycine buffer (pH 7.4), followed by mixing and incubation at room temperature for 20 min. The mixture was centrifuged at 9170 ***g*** for 30 min at 4°C, and the supernatant was isolated as BDS. HDL-cholesterol (HDL-C) concentrations in serum were determined by measuring total cholesterol (TC) concentrations in BDS sample. HDL protein concentrations in BDS and serum were determined using the protein-to-cholesterol ratio obtained from the same individual’s HDL, which was isolated by ultracentrifugation as described in the following section.

### Isolation of HDL

HDL (d = 1.063–1.210 g/ml) was isolated from pooled serum by ultracentrifugation as described previously [18]. The isolated HDL was dialyzed against the PON1 buffer (2 mM CaCl_2_, 50 mM Tris-HCl, pH 7.4) or phosphate-buffered saline. The samples were stored at −80°C until use in all analyses. TC concentrations were measured using an enzymatic test kit (Denka Co. Ltd., Tokyo, Japan). Total protein (TP) concentrations were assayed using the Folin–Lowry method [19].

### Preparation of purified apoA-I and rHDL

To delipidate HDL, the HDL derived by ultracentrifugation was dripped into ethanol:diethyl ether (3:2, v/v) with stirring and left overnight at −20°C. Then, the insoluble precipitate was collected. Diethyl ether was subsequently added, and the mixture was kept overnight at -20°C to further delipidate the protein. The delipidated protein, containing apoA-I, was then collected. Dried precipitate was dissolved in 6.8 M Urea, 10 mM Tris-HCl, pH8.0. ApoA-I was purified from the HDL protein solution by size exclusion chromatography (SEC) with Sephacryl S-200 HR, at a flow rate of 0.2 ml/min. Fractions that contained apoA-I abundantly were picked up and dialyzed against 20 mM Tris-HCl, pH7.4.

Preparation of rHDL followed a previously reported method with minor modifications [20-22]. First, lecithin and free cholesterol were individually dissolved in methanol:chloroform (3:1, v/v) at the specified ratios. The solvent was dried by N_2_ gas, and sodium deoxycholate dissolved in TEN buffer (150 mM NaCl, 1 mM EDTA-2Na, 10 mM Tris-HCl, pH8.0) was added. The concentration of sodium deoxycholate was adjusted such that the molar ratio of total lipid to deoxycholate was 1:1.2. The mixture was vortexed for three min and incubated for 15 min at 37°C. This step was repeated five times. Subsequently, purified apoA-I or 4.0 mM Hcy-thiolactone treated apoA-I solution and the derived lipid solution were mixed and incubated at 37°C with 600 rpm for 1 h. To generate several sizes of rHDL particles, the molar ratios of apoA-I:free-cholesterol:egg lecithin were set at 1:1.8:34, 1:4.7:140, respectively [21]. To remove sodium deoxycholate, the resultant was dialyzed against TEN buffer at 4°C with three buffer exchanges during three days. The rHDL particles were isolated from each resultant by SEC with Sephacryl S-200 HR at a flow rate of 0.2 ml/min. Finally, we selected fractions which contained different sizes of rHDL particles and collected them as small, medium, and large rHDL, respectively. These were then concentrated with Amicon Ultra-0.5 ml Centrifugal Filters (pore size: 10 kDa) (Merck Millipore Ltd. Germany) for subsequent experiments.

### *N*-homocysteinylation of specimens

*N*-homocysteinylation of specimens was performed according to a previously reported method with minor modifications [23,24]. Briefly, dl-homocysteine thiolactone hydrochloride (Fujifilm Wako Pure Chemicals Corporation, Osaka, Japan) was dissolved in 2 mM CaCl_2_, 50 mM Tris-HCl, pH 10.4 as a stock solution. The pH of the final reaction mixture was confirmed to be neutral (e.g., pH7.4) using pH test paper before use for experiments. For *N*-homocysteinylation reactions, HDL isolated from pooled healthy serum (4 mg proteins/ml), serum (45 mg HDL-C/dl), human purified PON1 (rePON1, 0.2 mg/ml), purchased from NKMAX Co., Ltd. (Korea), purified apoA-I (0.3 mg/ml), apoA-I with rePON1, and rHDL with rePON1 (0.1 mg apoA-I/ml and 0.2 µM rePON1) were incubated with various concentrations of Hcy-thiolactone (e.g., 0.8, 1, 1.5, 2, 4, 10, and 20 mM) at 37°C for 24 h. Samples were not dialyzed prior to activity measurement, and thus contained free Hcy-thiolactone. This approach also helped avoid protein loss.

### PON1 arylesterase activity

Arylesterase activity was determined following a previously described method with minor modifications [25]. Briefly, 13 µl of sample was mixed with 57 µl of arylesterase buffer (1.3 mM CaCl_2_, 100 mM Tris-HCl, pH 8.0) and 30 µl of 2 mM p-nitrophenyl acetate (Fujifilm Wako Pure Chemicals Corporation, Osaka, Japan) dissolved in 90 mM Tris-HCl (pH 8.0). Initial velocities of hydrolysis were determined at 405 nm. The E_405_ for the reaction was 18,050 ml/mmol/cm.

### PON1 paraoxonase activity

Paraoxonase activity was determined by following a previously described method [26]. Briefly, 40 µl of sample was mixed with 30 µl of paraoxonase buffer (2.63 M NaCl, 1.3 mM CaCl_2_, 50 mM Tris-HCl, pH 8.5) and 30 µl of 2 mM paraoxon (Toronto Research Chemicals Inc., Toronto, Canada) dissolved in 50 mM Tris-HCl (pH 8.5). Initial velocities of hydrolysis were determined at 405 nm. The E_405_ for the reaction was 18,050 ml/mmol/cm.

### PON1 thiolactonase activity

Thiolactonase activity was determined following a previously described method [27]. Briefly, 20 µl sample was mixed with 40 µl of 1 mM 5,5’-Dithiobis-(2-nitrobenzoic Acid) (Fujifilm Wako Pure Chemicals Corporation, Osaka, Japan) and 40 µl of 50 mM γ-thiobutyrolactone (Sigma-Aldrich Japan LLC, Tokyo, Japan) in the PON1 buffer (2 mM CaCl_2_, 50 mM Tris-HCl, pH 7.4). Initial velocities of hydrolysis were determined at 405 nm. The E_405_ for the reaction was 14,150 ml/mmol/cm.

### Western blot analysis

Western blotting (WB) was conducted following a previously described method. Serum IgG was depleted using protein G beads (Sigma-Aldrich Japan LLC, Tokyo, Japan) to decrease nonspecific bindings. For SDS-PAGE, uHDL (1 µg HDL protein/ml) and serum samples (0.07 µg HDL protein/ml) were loaded onto a 12.5% sodium dodecyl sulfate polyacrylamide gel. For Native-PAGE, uHDL (5 µg HDL protein/ml) and serum samples (0.34 µg HDL protein/ml) were analyzed using a 7% nondenaturing polyacrylamide gel. As the concentration of PON1 proteins in serum is higher than in uHDL samples, diluted serum samples were used for this analysis. Separated proteins were transferred to PVDF membranes (Millipore, Massachusetts, U.S.A.). The membranes were blocked in 5% skim milk at room temperature for 1 h. Then, the membranes were washed with tris buffered saline with tween 20 (TBST) and incubated overnight at 4°C with primary antibodies: monoclonal rabbit anti-PON1 antibody (1:5000) and polyclonal goat anti-apoA-I antibody (1:2000, 5000). After washing three times with TBST, the membranes were incubated with HRP-conjugated secondary antibodies (1:50000): polyclonal goat anti-rabbit IgG and polyclonal rabbit anti-goat IgG at room temperature for 1 h. After additional washes with TBST, the bands were visualized using ECL Prime Western Blotting Detection Reagents (GE Healthcare, Tokyo, Japan) or 3,3′- diaminobenzidine tetrahydrochloride (DAB) and hydrogen peroxide. Semiquantification of each band and molecular mass determination were performed by densitometry on a CS Analyzer 4 (ATTO, Tokyo, Japan).

### MALDI-TOF MS analysis

Proteins were dissolved in 25 mM ammonium bicarbonate solution. Reduction was accomplished with the addition of 100 mM dithiothreitol (final conc. 5.5 mM). Protein solutions were thermally denatured by incubating at 95°C for 5 min. Carboxymethylation was achieved with the addition of 100 mM 2-iodoacetamide (IAA) (final conc. 10 mM). The reaction was carried out in the dark for 20 min at room temperature. Digestion was started with the addition of 1 µl of the 0.1 mg/ml trypsin solution. In solution, digestion was performed for samples at 37°C overnight using proteomics-grade trypsin (Sigma-Aldrich Japan LLC, Tokyo, Japan). A digestion solution was desalted using GL Tip SDB (GL Science Inc., Tokyo, Japan). For MALDI-TOF MS analysis, 1 µl of the obtained tryptic peptides and equal volume 2,5-dihydroxybenzoic acid (DHB) matrix solution (10 mg/ml DHB in water:acetonitrile, 1:1 v/v containing 0.3% trifluoroacetic acid) were mixed in a 1.5 ml microcentrifuge tube. Then, 2 μl of the mixture was spotted on MTP 384 target plate (Bruker Co., MA U.S.A.) and left to dry at room temperature. MALDI-TOF MS was performed using an UltrafleXtreme MALDI-TOF mass spectrometer (Bruker). The mass spectra were acquired over the m/z range of 300–5,000 in Reflectron Positive ion modes (RP mode). Tandem mass spectrometry (MS/MS) analysis was performed using LIFT mode. The data obtained was processed using BioTools (version 3.2, Bruker) and searched using Mascot search algorithm (version 2.4) against a human protein database (Swiss-Prot database) modified to include *N*-homocysteinylation at lysine residues. Amino acid residue numbering corresponds to the mature form of human apoA-I (243 amino acids) after cleavage of the 18-amino acid signal peptide and 6-amino acid propeptide. The first amino acid of the mature apoA-I protein is designated as position 1.

### Cholesterol efflux capacity measurement

Choelsterol efflux capacity (CEC) of *N*-homocysteinylated pooled healthy serum was evaluated by immobilized liposome-bound gel beads (ILG) method. ILGs were prepared as described previously [28]. Liposomes were formed by evaporating the chloroform containing 10.6 mg of lecithin, 2.3 mg of cholesterol, and 0.0075 mmol of 23-(dipyrrometheneboron difluoride)-24-norcholesterol (BODIPY-labeled cholesterol; Avanti Polar Lipids Inc., Alabaster, AL, U.S.A.) under a stream of N_2_ gas. The step that diethyl ether was added to the resultant and was evaporated was repeated twice. Derived lipid film was suspended in 14 ml of 10 mM Tris-HCl (pH 7.4) containing 150 mM NaCl and 1 mM Na_2_EDTA (Buffer A), and BODIPY-cholesterol containing liposome was formed. The liposome solution and 0.7 g of Sephacryl S 300 HR were mixed, and the mixture was frozen at −80°C and thawed at 25°C seven times. Liposomes that were not incorporated as ILG were washed out, and obtained ILGs were stored in 10 ml of Buffer A in the dark at 4°C until use. For CEC evaluation, specimens were dialyzed against PON1 buffer in advance to avoid interference of CEC measurement by Hcy-thiolactone. Dialyzed specimen was diluted to achieve the same cholesterol concentration. Treated specimens (150 µl) were mixed with 100 µl of ILG with a setting corresponding to a final serum concentration of 2% (v/v) based on the samples’ cholesterol concentration. The mixtures were incubated for 16 h at 25°C in the dark. After spinning down ILGs, 75 µl of supernatant was collected and transferred to a Corning® 96-well Half Area Black Flat Bottom Polystyrene Not Treated Microplate (Corning Inc., NY, U.S.A.). Fluorescence intensity (excitation wavelength: 485 nm, emission wavelength: 538 nm) was measured using a Fluoroskan Ascent (Thermo Fisher Inc., Tokyo, Japan).

### Statistical analysis

We used one-way analysis of variance with Tukey correction or Games-Howell correction to compare the results. The results are expressed as means ± SD. A *P*<0.05 was considered statistically significant. Comparisons of PON1 activities between the rHDL consisting of untreated and treated apoA-I with Hcy-thiolactone were performed using paired *t*-tests.

## Results

### Mass spectrometry confirmation of apoA-I *N*-homocysteinylation and absence on PON1

We investigated whether Hcy-thiolactone directly modified the PON1 protein by MALDI-TOF MS. Initially, we confirmed that purified apoA-I was indeed modified by Hcy-thiolactone. Its modification sites were identified through peptide mapping analysis; specifically, upon searching for *N-*homocysteinylated lysine residues (*N-*Hcy-Lys), *N-*Hcy-Lys_83_ of apoA-I was identified after 0.8 mM Hcy-thiolactone treatment (**Table 1**). Furthermore, multiple modification sites (Lys_69, 83, 131, 164_) were detected when treated with higher concentrations of Hcy-thiolactone treatment. The peptide coverage rates were 89% and 83% for 0.8 and 4.0 mM Hcy-thiolactone treatments, respectively. However, no Hcy-thiolactone-modified sites were detected in rePON1 protein after treatment with 2 and 10 mM Hcy-thiolactone, even though the adequate peptide coverage rates (73% and 75%, respectively) were achieved. A mixture of rHDL and rePON1 was also analyzed to investigate PON1 modification within an HDL-like environment, as PON1 anchored to HDL particles *in vivo*. Even though *N-*Hcy-Lys_131_ of apoA-I was detected in rHDL samples, no modification sites of PON1 on rHDL were observed (peptide coverage rate: 87% of apoA-I, and 62% of PON1). Detailed mass spectra and mass spectrometry results of fragments containing *N-*Hcy-Lys in apoA-I were shown in the online supplementary figures 1-3, and online supplementary tables 1-7. These results demonstrated that PON1 protein does not undergo direct *N*-homocysteinylation, unlike apoA-I.

**Table 1:** *N*-Hcy-peptides identified by MALDI-TOF MS modified with Hcy-thiolactone

apoA-I *N*-Hcy-peptides	*N*-Hcy-Lys residue	Strat-end
Purified apoA-I with 0.8 mM HcyT		
LLDNWDSVTSTFSK^Hcy^ LR	83	70–85
Purified apoA-I with 4.0 mM HcyT		
DSGRDYVSQFEGSALGKQLNLK^Hcy^	69	48–69
DYVSQFEGSALGKQLNLK^Hcy^	69	52–69
LLDNWDSVTSTFSK^Hcy^ LR	83	70–85
K^Hcy^ WQEEMELYR	131	131–140
LHELQEK^Hcy^ LSPLGEEMR	164	158–173
rHDL with 1.5 mM HcyT		
K^Hcy^ WQEEMELYR	131	131–140

K^Hcy^ (underline) denotes *N*-Hcy-Lys residue.

apoA-I, apolipoprotein A-I. HcyT, homocysteine thiolactone. rHDL, reconstituted HDL.

### Regulation of PON1 activity by HDL particle size

**Figure 1:**
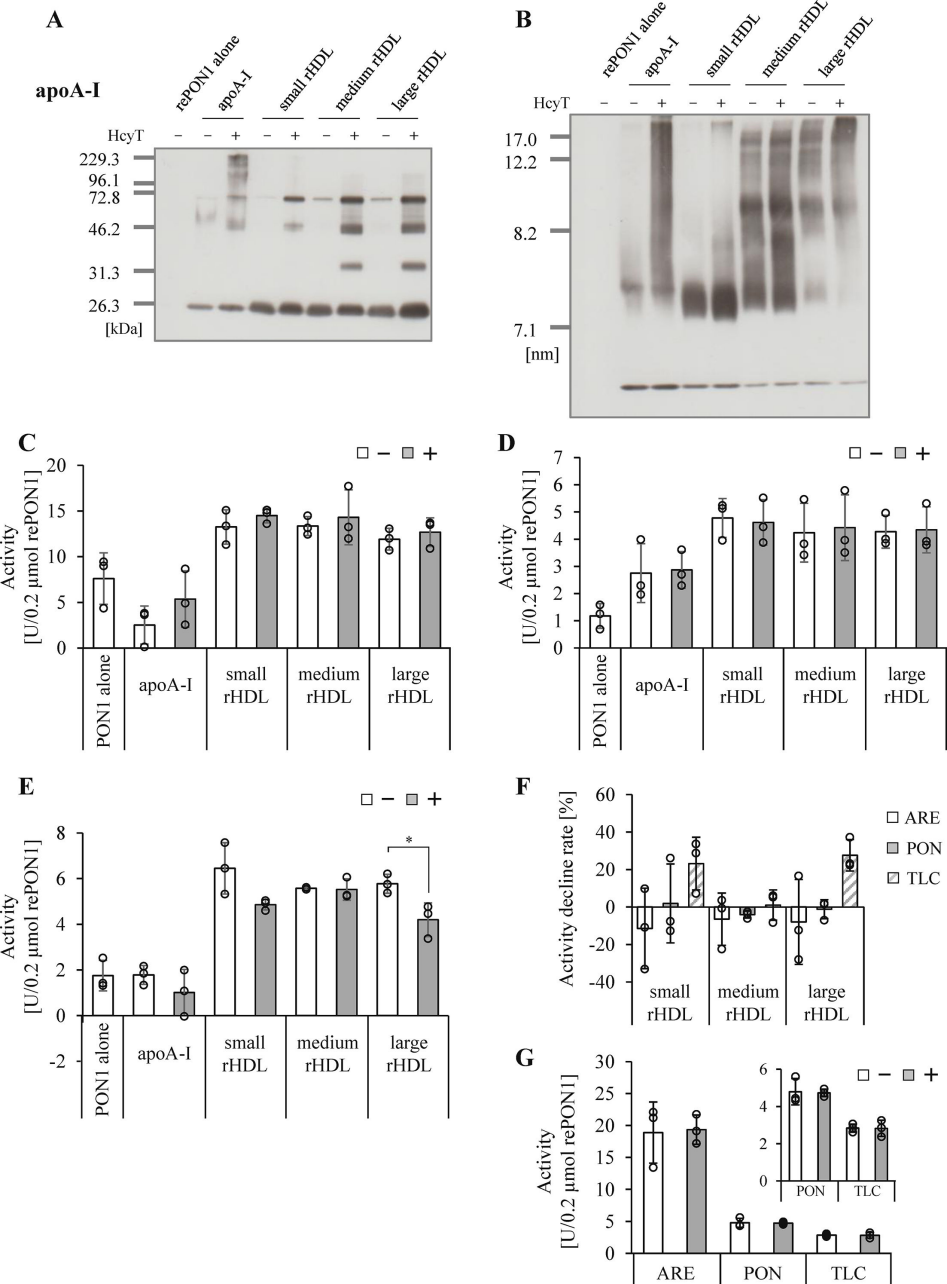
Effect of Hcy-thiolactone treatment on PON1 activity in various sizes of rHDL. Representative Western blot profiles of apoA-I by SDS-PAGE (0.3 µg apoA-I/lane) (**A**) and Native-PAGE (0.5 µg apoA-I/lane) (**B**). PON1 activities were measured in sample without (□) or with (■) HcyT: arylesterase activity (**C**); paraoxonase activity (**D**); thiolactonase activity (**E**) and decline ratio of activities was shown (**F**). Samples were mixtures of rePON1 (0.2 µM) and rHDL or purified apoA-I (0.1 mg/ml apoA-I) incubated without or with 1.5 mM HcyT at 37°C for 24 h. PON1 activities are expressed per 0.2 µmol rePON1. Samples were mixtures of rePON1 (0.2 µM) and rHDL composed of apoA-I pre-treated without (□) or with (■) HcyT (0.1 mg/ml apoA-I) and then incubated at 37°C for 24 h. PON1 activities are expressed per 0.2 µmol rePON1(G). Representative profiles from two independent experiments are shown (**A, B**). Data (**C-G**) are presented as mean ± SD from three independent experiments, with duplicate measurements performed for each sample within each experiment (**C-G**). *P<0.05 determined by paired *t*-test. apoA-I, apolipoprotein A-I; ARE, arylesterase activity; HcyT, homocysteine-thiolactone; PON1, paraoxonase 1; PON, paraoxonase activity; rHDL, reconstituted HDL; TLC, thiolactonase activity.

Given that PON1 was not directly modified by Hcy-thiolactone, we hypothesized that *N*-homocysteinylated apoA-I could influence PON1 activity. To test this, we prepared different sizes of rHDL composed of varying concentrations of protein, phospholipids (PL), and cholesterol (online supplementary figure 4, online supplementary table 8). We then incubated these rHDL preparations, or purified apoA-I, with or without Hcy-thiolactone. First, we confirmed that neither purified apoA-I nor rHDL possessed intrinsic activities (arylesterase, paraoxonase, and thiolactonase) associated with PON1 as shown in online supplementary table 9. Next, we analyzed structural change in apoA-I and PON1 proteins after Hcy-thiolactone treatment using Western blotting. Hcy-thiolactone treatment promoted the formation of apoA-I complexes in rHDL, and the apoA-I complex levels in medium and large rHDL were higher than those in small rHDL (**Figure 1A**). Although a previous study reported that apoA-I treated with Hcy-thiolactone exhibited higher lipid-binding capacity and reconstituted larger HDL particles than untreated apoA-I [7], our Hcy-thiolactone treatment did not affect the overall HDL particle sizes in rHDL preparations (**Figure 1B**). Next, we measured three types of PON1 activities in these samples. Interestingly, PON1 activities (arylesterase, paraoxonase, and thiolactonase) were consistently higher when rePON1 was incorporated into rHDL particles compared with rePON1 alone, despite the inclusion of the same amount of rePON1. In addition, PON1 activities in rHDL without Hcy-thiolactone did not significantly change across various rHDL sizes, suggesting that the size distribution of PON1 on HDL particles does not independently affect PON1 activities. Regarding the effect of Hcy-thiolactone treatment, when apoA-I was homocysteinylated in the presence of rePON1, it did not significantly modulate any of the three PON1 activities (arylesterase, paraoxonase, and thiolactonase) (**Figure 1C–E**). Furthermore, when rePON1 was incorporated into rHDL particles, *N*-homocysteinylated apoA-I did not significantly alter arylesterase and paraoxonase activities across all sizes of rHDLs (**Figure 1C and D**). Conversely, thiolactonase activity on large rHDL significantly decreased with Hcy-thiolactone treatment, and a tendency for lower activity was also observed in small rHDL compared with untreated rHDL (**Figure 1E**). Of note, thiolactonase activity on medium rHDL was not affected by Hcy-thiolactone. Comparing the rate of activity reduction by Hcy-thiolactone treatment, PON1 activities in medium rHDL were not significantly affected, but only thiolactonase activity was decreased in large and small rHDL (**Figure 1F**). The reduction of PON1 activity by Hcy-thiolactone may be affected differently depending on the HDL particle size.

To specifically investigate the direct effect of apoA-I modification on PON1 activity, we compared PON1 activities in large rHDL composed of apoA-I with or without Hcy-thiolactone treatment. All three types of PON1 activity remained comparable regardless of the apoA-I modification (**Figure 1G**). ApoA-I modification with 4.0 mM Hcy-thiolactone was confirmed by mass spectrometry (**Table 1**). The particle sizes and PL/TP ratios of the rHDL were similar between the untreated and modified apoA-I preparations (online supplementary figure 5, and PL/TP values: 10.3 for untreated, 10.9 for modified). These results suggest that *N*-homocysteinylation of apoA-I does not directly inhibit PON1 enzyme activity.

### Effect of Hcy-thiolactone treatment on PON1 and HDL particles in uHDL

**Figure 2:**
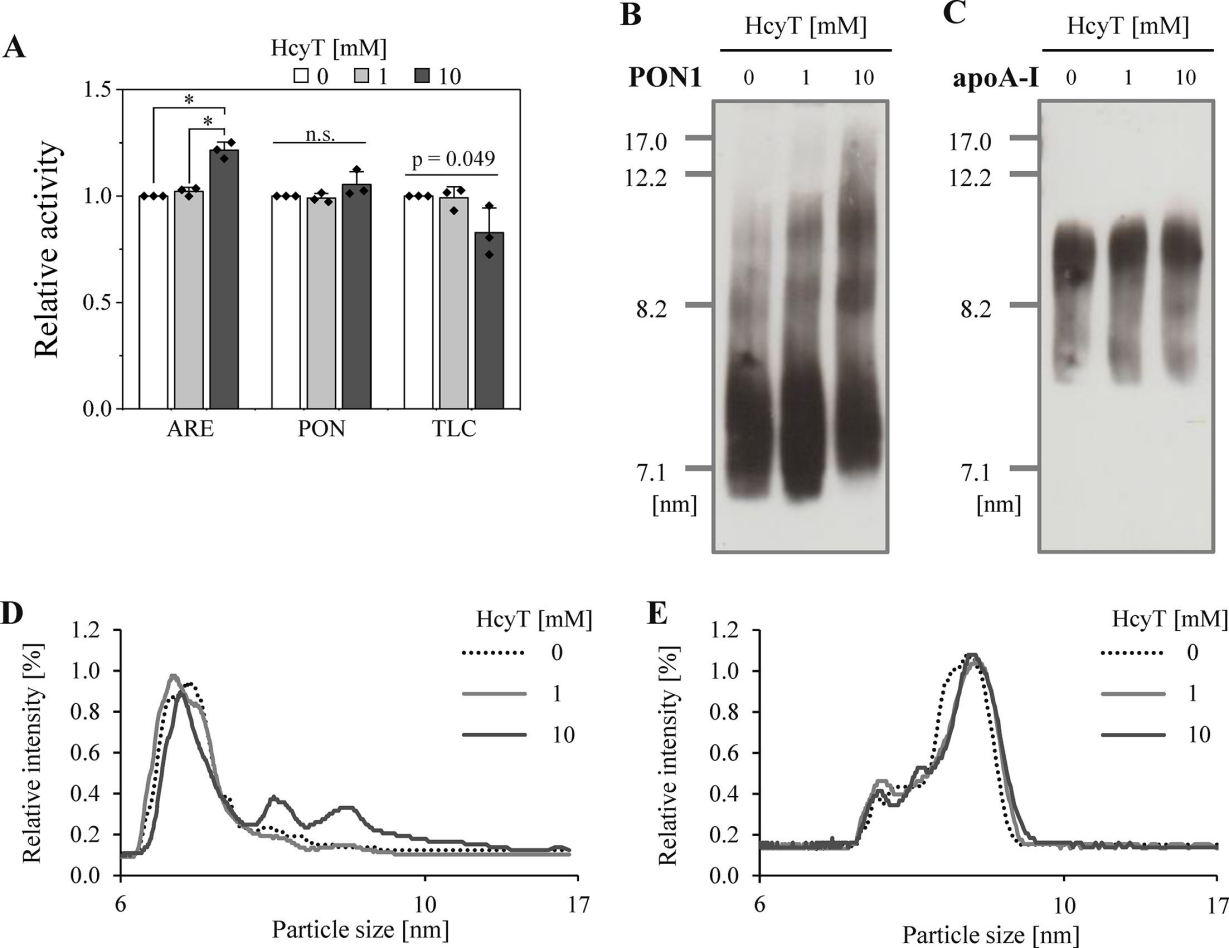
Analysis of PON1 activity and distribution in uHDL samples with Hcy-thiolactone treatment. (**A**) Relative PON1 activities in uHDL samples with HcyT treatment: arylesterase (ARE) activity; paraoxonase (PON) activity; thiolactonase (TLC) activity. Representative Western blots with densitometric quantification graphs of PON1 (**B, D**) and apoA-I (**C, E**) by Native-PAGE (5.0 µg protein/lane). Samples were uHDL (4 mg/ml) incubated with 0, 1, and 10 mM HcyT at 37°C for 24 h. The semiquantitative results of Western blot analysis of PON1 and apoA-I are shown as a percentage of total intensity per lane, as Hcy-thiolactone tended to enhance ECL or DAB development. Samples were assayed in triplicate (**A**). Representative profiles from three independent experiments are shown (**B-E**). Data are presented as mean ± SD from three independent experiments (**A**). *P<0.05 determined by one-way analysis of variance with Tukey correction or Games–Howell correction. apoA-I, apolipoprotein A-I; HcyT, homocysteine-thiolactone; n.s., not significant; uHDL, HDL fraction isolated by ultracentrifugation; PON1, paraoxonase 1.

PON1 binds to various proteins associated with HDL and its activities are modulated by these proteins, such as apoA-I and apolipoprotein A-II, and myeloperoxidase [29-32]. To further investigate the effect of Hcy-thiolactone on PON1 associated with native HDL, we treated uHDL samples with Hcy-thiolactone and analyzed PON1 activity and protein modifications by enzymatic assay and immunoblotting, respectively. Unexpectedly, 10 mM Hcy-thiolactone treatment significantly increased arylesterase activity by 20% compared with untreated samples. In contrast, paraoxonase activity remained similar to that in untreated uHDL, and thiolactonase activity tended to be suppressed in 10 mM Hcy-thiolactone treated HDL (**Figure 2A**). These observed modulations in PON1 activities suggested potential alterations in PON1’s association with HDL particles. To investigate this possibility, we further examined the distribution of PON1 on HDL by Native-PAGE. Treatment of 10 mM Hcy-thiolactone reduced the amount of PON1 on the HDL particle of approximately 7 nm size. Concurrently, there was a tendency for the PON1 distribution to shift toward larger HDL particles (8–10 nm) (**Figure 2B and D**). This observed shift was specific to PON1 protein, as the distribution of apoA-I remained unchanged (**Figure 2C and E**), indicating that the overall HDL particle size distribution was not altered.

**Figure 3:**
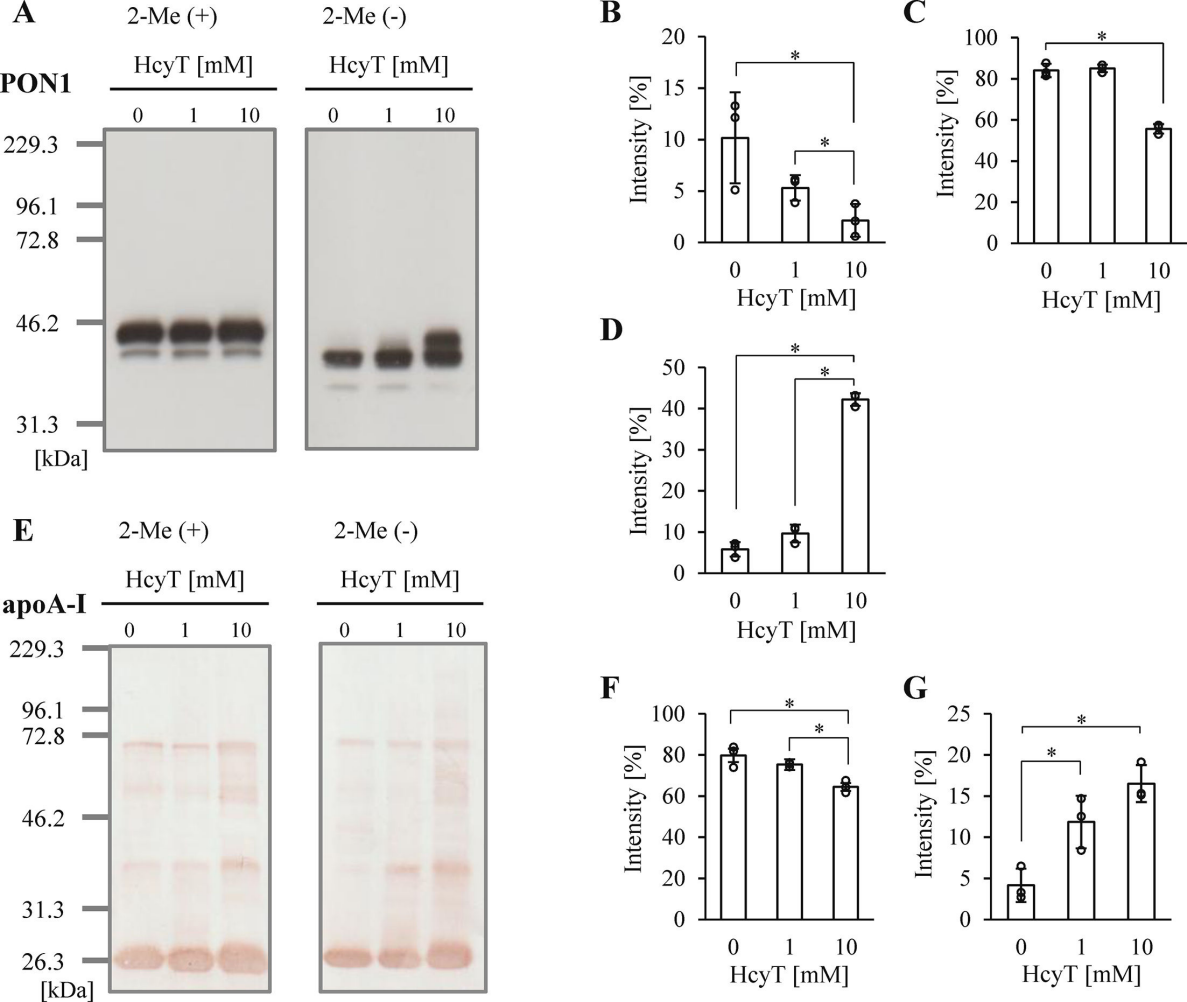
SDS-PAGE analysis of PON1 and apoA-I proteins in uHDL samples with Hcy-thiolactone treatment. (**A**) Representative Western blots of PON1 by SDS-PAGE (1.0 µg protein/lane) under reducing (2-Me(+)) (left panel) and nonreducing (2-Me(-)) (right panel) conditions. Quantification analysis of PON1 bands shown as percentage of total intensity at approximately 37 (**B**), 42 (**C**), and 45 kDa (**D**) without reduction. (**E**) Representative Western blots bands of apoA-I by SDS-PAGE (1.0 µg protein/lane) under reducing (2-Me(+)) (left panel) and nonreducing (2-Me(-)) (right panel) conditions. Quantification analysis of apoA-I bands shown as percentage of total intensity at approximately 25 (**F**) and 38 kDa (**G**) without reduction. Samples were uHDL (4 mg/ml) incubated with 0, 1, and 10 mM HcyT at 37°C for 24 h. Representative profiles from three independent experiments are shown (**A, E**). Data are presented as mean ± SD (**B-D, F-G**). *P<0.05 determined by one-way analysis of variance with Tukey correction or Games–Howell correction. 2-Me, 2-mercaptoethanol; apoA-I, apolipoprotein A-I; HcyT, homocysteine thiolactone; uHDL, HDL fraction isolated by ultracentrifugation; PON1, paraoxonase 1.

To evaluate complex formation of HDL proteins, we further analyzed the protein profile of HDL after Hcy-thiolactone treatment by SDS-PAGE. Regarding PON1, two intact bands were observed in the untreated sample under reducing conditions (**Figure 3A**), consistent with previous reports [33], as PON1 is a glycoprotein. Additionally, under nonreducing conditions, PON1 exhibited an apparent molecular mass of approximately 42 kDa, which increased to 46 kDa upon reduction, consistent with its known disulfide-bridged structure. Under reducing conditions, no significant changes were observed in intact PON1 bands (43 and 46 kDa) following treatment with either 1 mM or 10 mM Hcy-thiolactone (**Figure 3A**, online supplementary figure 6AB). In contrast, under nonreducing conditions, a band with an apparent molecular mass of approximately 45 kDa was observed after 10 mM Hcy-thiolactone treatment (**Figure 3D**). Concurrently, the bands at approximately 37 kDa (**Figure 3B**) and 42 kDa (**Figure 3C**) significantly decreased with 10 mM Hcy-thiolactone treatment. Regarding the analysis of apoA-I, some bands at higher molecular weights were observed under both reducing and nonreducing conditions (**Figure 3E**). Although the intensity of the intact band (25 kDa) was significantly lower in 10 mM Hcy-thiolactone treated HDL under reduction condition, complex formation did not change with HcyT treatment compared with the untreated sample (**Figure 3E**, online supplementary figure 6C-F). On the other hand, under nonreducing conditions, the percentage of intensity at 25 kDa was decreased, and that at 38 kDa was significantly increased by 10 mM Hcy-thiolactone compared with those of untreated samples (**Figure 3F and G**).

### Impact of Hcy-thiolactone treatment on PON1 in serum samples

**Figure 4:**
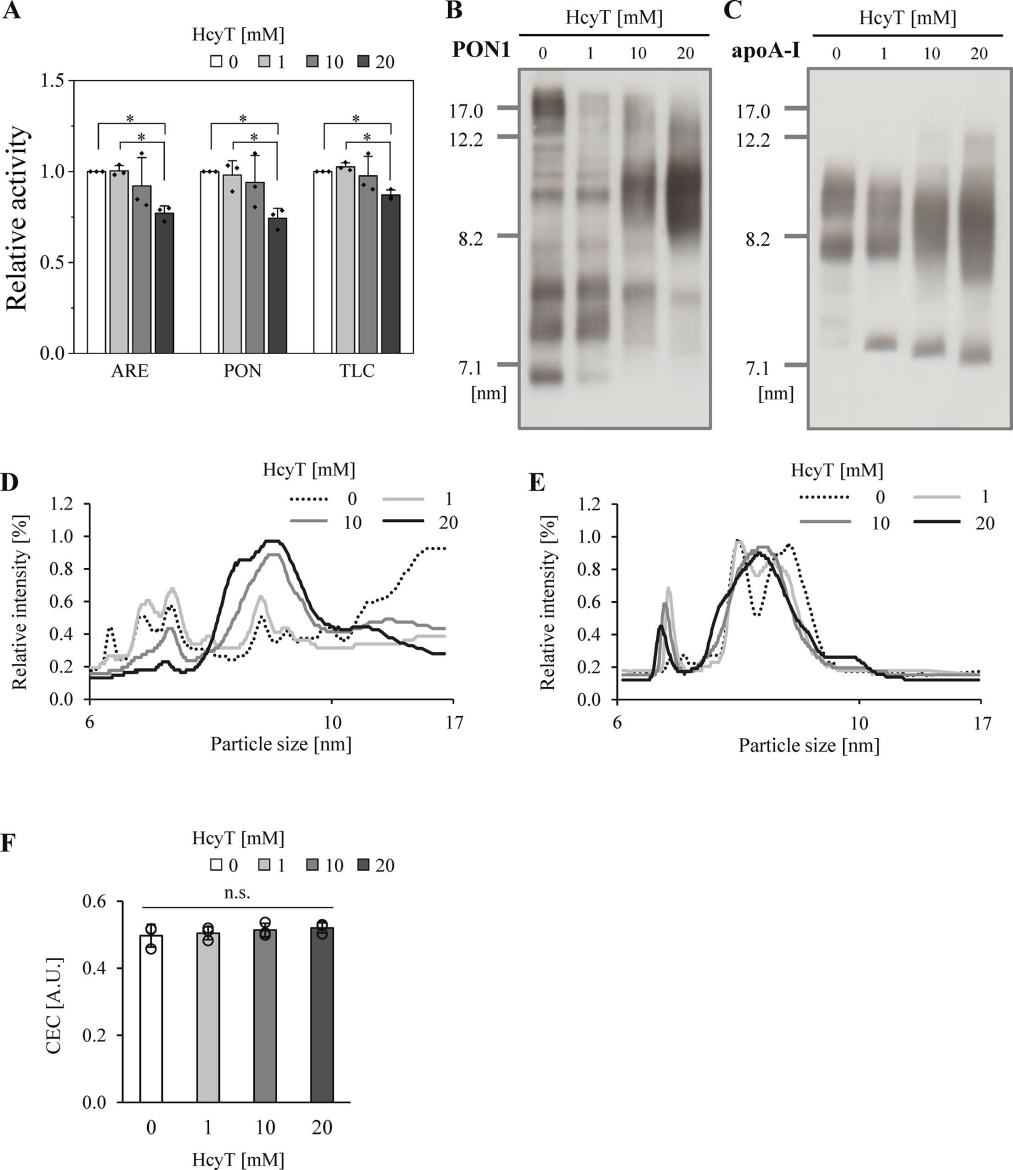
Analaysis of PON1 activity and distribution in serum samples with Hcy-thiolactone treatment. (**A**) Relative PON1 activities in serum samples with HcyT treatment: arylesterase (ARE) activity; paraoxonase (PON) activity; thiolactonase (TLC) activity. Representative Western blots with densiometric quantification graphs of PON1 (**B, D**) and apoA-I (**C, E**) by Native-PAGE (0.34 µg HDL protein/lane). Cholesterol efflux capacity in serum samples with HcyT treatment (**F**). Samples were serum (45 mg HDL-C/dl) incubated with 0, 1, 10, and 20 mM HcyT at 37°C for 24 h. Samples were assayed in triplicate (**A, F**). Representative profiles from three independent experiments are shown (**B-E**). Data are presented as mean ± SD from three independent experiments (**A, F**). *P<0.05 determined by one-way analysis of variance with Tukey correction or Games–Howell correction. apoA-I, apolipoprotein A-I; CEC, cholesterol efflux capacity; HcyT, homocysteine thiolactone; n.s.; not significant; PON1, paraoxonase 1.

To further investigate the effects of Hcy-thiolactone on PON1 in whole serum samples, representing a more *in vivo*-like environment, we performed enzymatic assays and protein analyses. In these samples, all three types of PON1 activities were suppressed by approximately 15–25% following 20 mM Hcy-thiolactone treatment, a result differing from those observed in uHDL (**Figure 4A**).

To ensure that the observed activity changes were not due to direct interference from free Hcy-thiolactone or its degradation products in the assay, we performed a direct interference test. When PON1 activities were measured immediately after Hcy-thiolactone addition to serum, no concentration-dependent changes in activity were observed (online supplementary figure 7). This demonstrates that Hcy-thiolactone does not directly interfere with the substrate hydrolysis reaction or the detection system. Importantly, all primary comparisons were performed between treated and untreated samples within the identical specimen matrix, using the same buffer and assay protocols. The only variable was the presence of Hcy-thiolactone during the incubation period.

These results indicate that the observed reduction in PON1 activities is not an assay artifact but reflects the genuine structural and functional impact of apoA-I modification by Hcy-thiolactone. Given the observed changes in PON1 activity, we further examined the PON1 distribution using Native-PAGE. Hcy-thiolactone treatment significantly altered the PON1 distribution within serum (**Figure 4B**). While PON1 was initially distributed heterogeneously across various sizes of the HDL particles, Hcy-thiolactone treatment resulted in a translocation of PON1 towards 8.5–12 nm HDL particles (**Figure 4D**). Western blot analysis of apoA-I showed a bimodal distribution corresponding to HDL_2_ and HDL_3_ in untreated HDL. Upon treatment with 20 mM Hcy-thiolactone, the apoA-I distribution concentrated into an HDL size range of approximately 8 nm (**Figure 4C and E**). These results suggested that Hcy-thiolactone induced structural changes in HDL particles, leading to alterations in PON1 distribution, and that apolipoproteins including PON1 on HDL might have remodeling, causing the HDL population to become more uniform in size. These changes in distribution were maintained even after Hcy-thiolactone removal (online supplementary figure 8). These results suggested that protein modification by Hcy-thiolactone may contribute to the irreversible altered distribution. To assess the functional consequence of the altered PON1 distribution on anti-atherogenic capacity, we quantified CEC of serum treated with Hcy-thiolactone because CEC represents the critical anti-atherogenic function of HDL. Unexpectedly, no significant changes in CEC were observed in serum with Hcy-thiolactone treatment (**Figure 4F**).

**Figure 5:**
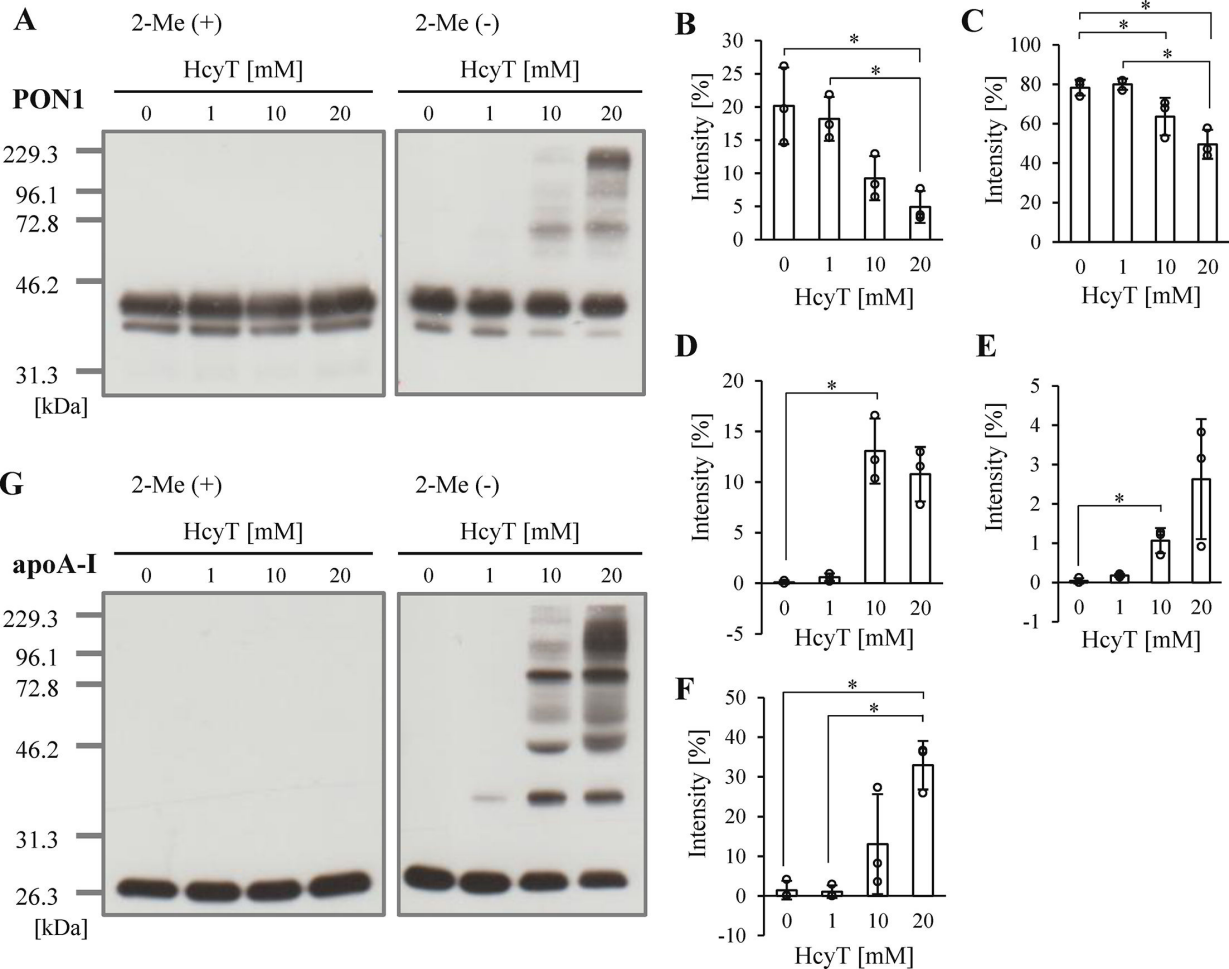
SDS-PAGE analysis of PON1 and apoA-I in serum samples with Hcy-thiolactone treatment. (**A**) Representative Western blots of PON1 by SDS-PAGE (0.07 µg HDL protein/lane) under reducing (2-Me(+)) (left panel) and nonreducing (2-Me(-)) (right panel) conditions. Quantification analysis of PON1 bands was shown as a percentage of total intensity at approximately 38 (**B**), 42 (**C**), 70 (**D**), 95 (**E**), and 135 kDa (**F**) without reduction. (**E**) Representative Western blots of apoA-I by SDS-PAGE (0.07 µg HDL protein/lane) under reducing (2-Me(+)) (left panel) and nonreducing (2-Me(-)) (right panel) conditions. Quantification analysis of PON1 bands is shown as a percentage of total intensity at approximately 38 (**B**), 42 (**C**), 70 (**D**), 95 (**E**), and 135 kDa (**F**) without reduction. Samples were serum (45 mg HDL-C/dl) incubated with 0, 1, 10, and 20 mM HcyT at 37°C for 24 h. Representative profiles from three independent experiments are shown (**A, G**). Data are presented as mean ± SD (**B-F**). *P<0.05 determined by one-way analysis of variance with Tukey correction or Games–Howell correction. 2-Me, 2-mercaptoethanol; apoA-I, apolipoprotein A-I; HcyT, homocysteine-thiolactone; PON1, paraoxonase 1.

Furthermore, we performed protein analysis by SDS-PAGE to investigate PON1 complex formation following HDL remodeling. Under reducing conditions, no changes in PON1 band patterns were observed following Hcy-thiolactone treatment (**Figure 5A**, online supplementary figure 9AB). Similar to the results obtained with uHDL samples, intact PON1 bands decreased following 10 mM Hcy-thiolactone treatment under nonreducing conditions (**Figure 5B and C**). However, unlike the results from uHDL samples, complexes with apparent molecular weights of approximately 70, 95, and 135 kDa were detected (**Figure 5D–F**). For apoA-I, complexes distinct from those observed in uHDL samples were also detected in serum treated with Hcy-thiolactone (**Figure 5G**, online supplementary figure 9C-F). These findings suggested that *N*-homocysteinylated apoA-I may bind to serum proteins or other proteins including PON1 present on HDL particles, which might be prone to detachment from HDL particles during ultracentrifugation, as indicated by the results from uHDL.

## Discussion

Higher homocysteine levels in blood are associated with various diseases including CVD. PON1 is one of the key enzymes for protection against CVD risk, and many epidemiological studies have demonstrated a strong inverse correlation between PON1 activities and CVD events [13,16]. Nonetheless, the effect of Hcy on PON1 remained unclear. Therefore, we aimed to investigate the effect of Hcy-thiolactone on PON1 activities and its structure.

Contrary to earlier suggestions that PON1 activity inhibition by Hcy-thiolactone was due to homocysteinylation of the PON1 protein [34], our results show no detection of Hcy-thiolactone-modified sites in rePON1, even after treatment with 10 mM Hcy-thiolactone. This concentration is considerably higher than those reported in previous studies that observed reduced PON1 activity, suggesting that direct modification of PON1 may not be the primary mechanism for its activity reduction.

In contrast, for apoA-I, the number of modified sites increased in an Hcy-thiolactone concentration-dependent manner. The specific modification sites of apoA-I were previously poorly characterized. Our MALDI-TOF MS analysis newly identified several modification sites (Lys_69, 83, 131, 164_) in purified apoA-I, whereas only *N*-Hcy-Lys_131_ was observed in rHDL samples. The identified Hcy-thiolactone modification sites were distinct from other lysine residues on apoA-I previously reported to undergo other chemical modifications. Although different chemical modifications may exhibit varying susceptibility, the underlying factors determining this susceptibility remain unclear. This homocysteinylation markedly promoted the formation of apoA-I complexes. Based on previous studies, the lipid binding capacity of apoA-I is also expected to be altered, suggesting potential changes in the particle structures of HDL containing *N*-homocysteinylated apoA-I.

The concentration of Hcy-thiolactone used in our *in vitro* assay (up to 10 mM) is substantially higher than the typical systemic physiological level (nmol/L range). However, this concentration was intentionally chosen as essential for the conclusive identification of the specific modification sites and the subsequent mechanistic dissection of functional changes. Furthermore, the physiological relevance of this concentration is supported by the fact that *N*-homocysteinylation is a known *in vivo* event, with approximately 1–7.4% of apoA-I reported to be modified even in healthy human serum [24]. More importantly, local concentrations of Hcy-thiolactone are known to be significantly elevated *in vivo*, particularly within cells (e.g. vascular endothelial cells) where it is actively generated, or in areas of severe pathology and inflammation where detoxification is compromised. Thus, our high concentration conditions may model these pathophysiologically relevant local environments necessary to initiate and study robust protein modification.

Given that the reduction in PON1 activity was not attributable to direct homocysteinylation of the PON1 protein, we hypothesized that *N*-homocysteinylated apoA-I or alterations in PON1’s location on HDL particles might be responsible for the observed decrease in PON1 activity by Hcy-thiolactone treatment. Indeed, PON1 activities differed significantly between lipid-free rePON1 and PON1 associated with rHDL (lipidated apoA-I), highlighting the importance of the lipid environment. ApoA-I binds to PON1 through two docking sites, which are crucial for stabilizing its activity and its binding to HDL particles [32,35]. In addition, it has been reported that the assembly of phospholipids into sufficiently large aggregates acted as an anchor for PON1 and thereby stabilized its activity. The PON1 binding stability with HDL particles is determined by HDL size and shape, which contributes to the amount of amphiphilic molecules, and it has been reported that approximately 100 detergent molecules are bound per PON1 monomer or dimer [36]. Both apoA-I and phospholipid contents of HDL contribute to the stabilization and docking of PON1 to HDL particles.

First, we examined the effect of Hcy-thiolactone on PON1 using rHDL because rHDL are simplified specimens only containing apoA-I, free cholesterol, and phospholipids. If *N*-homocysteinylated apoA-I directly destabilized PON1 activity, we would expect a direct correlation between the degree of apoA-I homocysteinylation and the extent of PON1 activity inhibition. However, our data showed no PON1 activity reduction in the mixture of apoA-I and rePON1 with Hcy-thiolactone treatment, and a difference in PON1 activity inhibition by Hcy-thiolactone between medium and large rHDL despite similar observations regarding apoA-I modification. This finding suggests that the suppression of PON1 activity by *N*-homocysteinylated apoA-I alone may not fully explain the observed effect. In addition, a decrease in activity due to a change in HDL particle size caused by *N*-homocysteinylated apoA-I could be denied, because HDL particle size did not change with or without Hcy-thiolactone treatment in both uHDL and serum. PON1 on large HDL was susceptible to reduced activity by Hcy-thiolactone. Furthermore, because PON1 on rHDL with the similar HDL size but differing only in apoA-I modification showed comparable PON1 activities with the notable exception of a significant decrease in thiolactonase activity specifically in large rHDL, apoA-I modification by Hcy-thiolactone does not directly affect PON1 activity. Changes in the lipid affinity of *N*-homocysteinylated apoA-I could affect PON1 activity by altering the phospholipids that anchor to PON1, and this effect may be more pronounced in lipid-rich HDL.

PON1 activity is also regulated by various proteins associated with HDL [29-32]. We verified the effects of Hcy-thiolactone on PON1 using uHDL. HDL is a highly heterogeneous lipoprotein, varying significantly in density, size, charge, shape, and especially in its lipid and protein composition. With nearly 100 proteins differentially associated, these proteins contribute to the distinct structure and functions of various HDL subclasses [37]. We could investigate the impact of Hcy-thiolactone on PON1 activity using uHDL, which includes more possible factors than with rHDL. Unexpectedly, arylesterase activity was increased in 10 mM Hcy-thiolactone treated uHDL, while thiolactonase activity tended to decrease by Hcy-thiolactone treatment. Therefore, thiolactonase activity may be susceptible to reduced activity by Hcy-thiolactone similar to rHDL. This discrepancy with a previous study, which reported a decrease in paraoxonase and lactonase activities upon Hcy-thiolactone addition in HDL samples [34], might stem from differences in the ultracentrifugation methods used for HDL isolation. The prior study’s method involved shorter ultracentrifugation times, potentially leading to less detachment of PON1 proteins from HDL particles. In line with this, we observed a notable difference in PON1 distribution in uHDL and serum samples. Furthermore, our data indicated that over half of the PON1 proteins were detached from HDL particles in our uHDL samples, as evidenced by PON1 activities per 1.0 mg/ml HDL protein in uHDL sample (Arylesterase, 152; paraoxonase, 84; thiolactonase, 36 U/l) being approximately half that in serum (Arylesterase, 493; paraoxonase, 201; thiolactonase, 186 U/l). In addition, our results demonstrated that Hcy-thiolactone treatment did not affect the overall HDL particle size in uHDL samples, as evidenced by the unchanged apoA-I distribution. Because HDL particles were unchanged and PON1 was not distributed in large HDL, a significant reduction in activity may not be observed. On the other hand, in uHDL treated with Hcy-thiolactone, immunoblotting analysis revealed that PON1 mobility was slightly reduced. This minimal change in apparent molecular mass (approximately 3 kDa) suggests that it is unlikely to be attributed to the covalent binding of other major apolipoproteins on HDL. Instead, this observation might reflect subtle conformational changes in PON1 or altered noncovalent interactions within the HDL particle.

While our experiments with pure HDL samples (rHDL and uHDL) provided insights into Hcy-thiolactone’s effect on PON1, we aimed to clarify these effects in a more *in vivo*-like environment. Therefore, we verified the effects of Hcy-thiolactone on PON1 remodeling and activity in serum samples. Our findings demonstrate that three types of PON1 activities were reduced and both apoA-I and PON1 distribution were notably affected by Hcy-thiolactone in serum samples. The remodeling of HDL due to the aforementioned changes in particle structures along with Hcy-thiolactone treatment may lead to PON1 distribution change.

Regarding the functional consequences, no significant changes in CEC were observed in serum treated with Hcy-thiolactone. This finding is notable and is supported by our ILG method, which provides results comparable with conventional cell-based assay [28]. Consistent with our findings, a previous study that measured CEC using the ILG method reported no significant differences in uHDL with or without Hcy-thiolactone treatment [38]. Furthermore, a molecular precedent established by Huang et al. [32] demonstrated that site-specific modification of apoA-I can lead to a total loss of PON1’s enzymatic and antioxidant activities without impairing the CEC of the HDL particle. Their structural and functional analyses revealed that the specific domains on apoA-I required for proper PON1 binding and activation are distinct and spatially decoupled from those required for CEC. Consequently, Hcy-thiolactone-induced modification of apoA-I can selectively impair the environment necessary for PON1 function while leaving the domains responsible for cholesterol efflux intact. Additionally, our results from the ILG method revealed that CEC was significantly affected by HDL particle size [38]. The Hcy-thiolactone-induced remodeling, which shifted the HDL distribution from a bimodal (large and small) population to a uniform, medium-sized population around 8 nm (**Figure 4C**), likely resulted in a compensatory offset of size-specific CEC variations. Thus, the decrease in CEC from the loss of large particles was balanced by the changes in the smaller/medium fractions, resulting in no net change in total CEC despite the dramatic structural changes. This consistency suggests that PON1 remodeling and activity reduction may not be immediately associated with a failure in the primary CEC of HDL. Therefore, we conclude that PON1 activity might serve as a more sensitive indicator of impairment by Hcy-thiolactone compared with bulk anti-atherogenic function such as CEC.

In contrast, in serum samples treated with Hcy-thiolactone, distinct high-molecular-weight complexes (approximately 70, 95, and 135 kDa) were observed. As PON1 was shown not to be directly modified by Hcy-thiolactone, the formation of these PON1 complexes is not due to an increase in thiol groups upon Hcy-thiolactone binding to PON1 itself. We propose that these bands represent abnormal covalent species formed through oxidative stress. Hcy-thiolactone is known to act as a pro-oxidant that generates reactive oxygen species in the presence of metal ions. This oxidative environment likely induces intermolecular disulfide bonds between PON1 and its partners. Specifically, the 70 kDa band likely represents a covalent apoA-I–PON1 complex, while the 95 and 135 kDa bands represent PON1 dimers and trimers, respectively. While further identification using immunoprecipitation–mass spectrometry would be beneficial in future studies, our current findings establish a clear link between Hcy-thiolactone-induced structural remodeling and the resulting biochemical dysfunction of PON1.

Considering the results in the current study, these complexes reflect a cascading process of structural reassociation rather than simple protein detachment. The sequence likely begins with the *N*-homocysteinylation of apoA-I, which alters its lipid affinity and triggers the structural remodeling of the HDL particle. This remodeling disrupts the specific hydrophobic microenvironment and the stabilizing interactions (including those with phospholipids) required to anchor PON1 in its native conformation. Our Native-PAGE analysis showed that after Hcy-thiolactone treatment, total PON1 band intensities in uHDL did not decrease, and free PON1 monomers did not increase in serum samples, suggesting that PON1 does not detach from the particle into a free state. Instead, the destabilized PON1 molecules likely undergo covalent aggregation via intermolecular disulfide bonds, potentially involving the free thiol group at cysteine 284 [9]. Since even aggregated PON1 (dimers and trimers) requires a hydrophobic environment for stabilization [36], these complexes likely remain associated or immediately reassociate with the remodeled HDL subpopulations. Furthermore, the shift from PON1 monomers to aggregates may alter the phospholipid requirement per PON1 molecule [36], potentially inducing a further release or reorganization of phospholipids. This synergistic effect between apoA-I modification and PON1 aggregation likely drives the dramatic shift in HDL particle size toward a uniform distribution. Therefore, the observed PON1 redistribution in serum reflects a sequence where apoA-I modification leads to HDL remodeling, followed by PON1 aggregation and structural reassociation with the remodeled particles.

One limitation of this study is that we cannot exclude the involvement of other apolipoproteins and serum proteins in modulating PON1 activity beyond what was observed in our controlled rHDL experiments. Regarding this point, further studies of rHDL with other proteins contributing to HDL structure, such as apoA-II and lecithin–cholesterol acyltransferase, would elucidate the mechanism of PON1 activity reduction by Hcy-thiolactone during changes in HDL remodeling *in vivo*. In this study, HDL remodeling was quantitatively characterized using Native-PAGE densitometry, a method established to correlate well with other biophysical measurements such as dynamic light scattering [39]. While additional orthogonal methods like nuclear magnetic resonance or electron microscopy would provide even more refined structural insights, our current results clearly establish the significant shift in HDL subpopulation distribution induced by Hcy-thiolactone. Nonetheless, our findings that specific rePON1 activities (e.g. thiolactonase) were affected by Hcy-thiolactone within rHDL (a simplified system containing only apoA-I, free cholesterol, and phospholipids) strongly suggest that the core HDL components themselves can influence PON1’s susceptibility to Hcy-thiolactone, independent of other complex serum proteins. In addition, a noteworthy observation was changes in the structural and distribution of PON1 in serum samples. The environment surrounding PON1, including lipids and apolipoproteins, is important in stabilizing PON1 and understanding the mechanism of PON1 dysfunction in pathological conditions. Further studies, such as analysis of clinical samples that reflect *in vivo* homocysteine concentrations, are needed to fully elucidate the complex interplay of these factors *in vivo* and their implications for CVD.

In conclusion, this study demonstrates that the reduction of PON1 activity induced by Hcy-thiolactone treatment was not attributed to *N*-homocysteinylation of PON1 protein, but is rather associated with *N*-homocysteinylation of apoA-I and subsequent alterations in HDL remodeling and PON1’s distribution on HDL particles. The distribution dynamics of PON1 in pathological conditions may help elucidate the mechanism of HDL function decline in CVD.

## Supplementary material

online supplementary figure 1

online supplementary table 1

## Data Availability

All data analyzed during this study are included in this article. The data used to support the findings of the present study are available from the corresponding author (Ryunosuke Ohkawa, Graduate School of Medical and Dental Sciences, Institute of Science Tokyo, ohkawa.alc@tmd.ac.jp) upon request.

## Abbreviations

ARE: arylesterase activity
BDS: apolipoprotein B-depleted serum
CEC: cholesterol efflux capacity
CVD: cardiovascular disease
DAB: 3,3′-diaminobenzidine tetrahydrochloride
DHB: 2,5-dihydroxybenzoic acid
ECL: enhanced chemiluminescence
HDL: high-density lipoprotein
HDL-C: HDL-cholesterol
HRP: horseradish peroxidase
Hcy: homocysteine
HcyT: Hcy-thiolactone
IAA: 2-iodoacetamide
ILG: liposome-bound gel beads
LDL: low-density lipoprotein
LOOHs: lipid hydroperoxides
MALDI-TOF MS: matrix-assisted laser desorption/ionization time-of-flight mass spectrometry
PEG: polyethylene glycol
PON1: paraoxonase 1
PON: paraoxonase activity
PVDF: polyvinylidene fluoride
SDS-PAGE: sodium dodecyl sulfate.polyacrylamide gel electrophoresis
SEC: size exclusion chromatography
TBST: Tris-buffered saline with Tween 20
TC: total cholesterol
TLC: thiolactonase activity
WB: Western blotting
apoA-I: apolipoprotein A-I
rHDL: reconstituted HDL
rePON1: recombinant PON1
uHDL: ultracentrifuged HDL
