# Supplementary material for: Homocysteine thiolactone affects paraoxonase 1 activity via altered paraoxonase 1 distribution on high-density lipoprotein particles
Source: Biochem J. 2026 Jan 22;46(Pt 1):BSR20253768. doi: 10.1042/BSR20253768 (PMC12905485; doi:10.1042/BSR20253768)
Supplement: online supplementary figure 1 [file bcj-46-1-BSR20253768-s001.pdf]

A

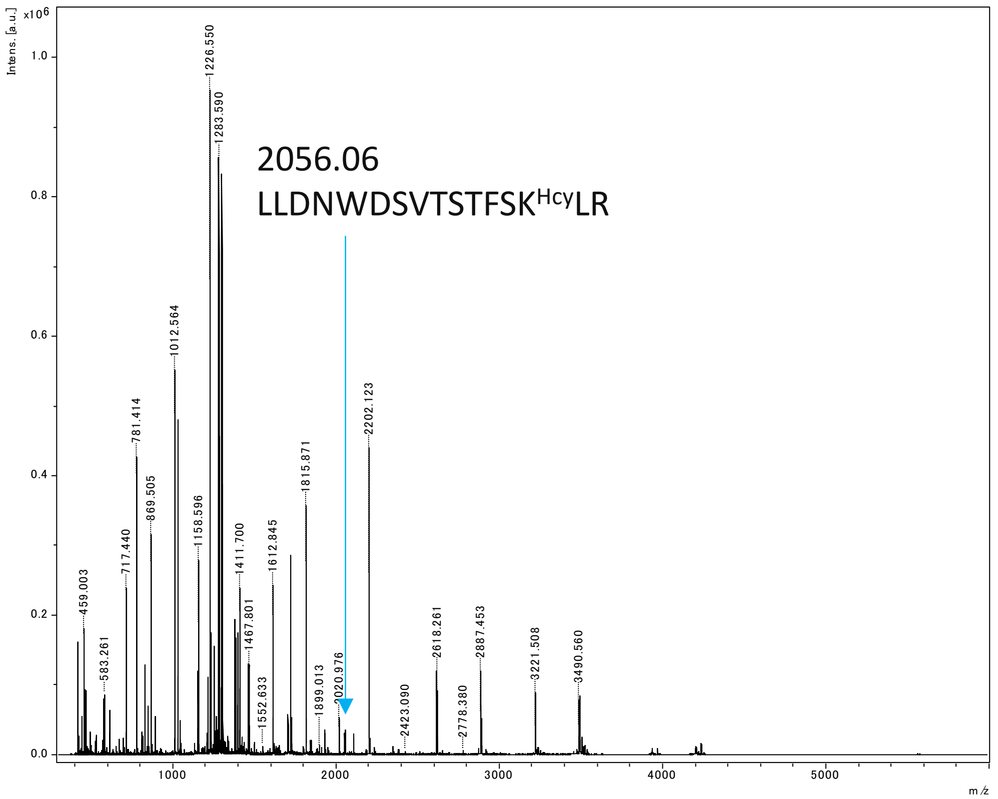

B

MKAAVLTLAV LFLTGSQARH FWQQDEPPQS PWDRVKDLAT 40
VYVDVLKDSG RDYVSQFEGS ALGKQLNLKL LDNWDSVTST 80
FSKLREQLGP VTQEFWDNLE KETEGLRQEM SKDLEEVKAK 120
VQPYLDDFQK KWQEEMELYR QKVEPLRAEL QEGARQKLHE 160
LQEKLSPLGE EMRDRARAHV DALRTHLAPY SDELRQRLAA 200
RLEALKENGG ARLAEYHAKA TEHLSTLSEK AKPALEDLRQ 240
GLLPVLESFK VSFLSALEEY TKKLNTQ 267

Supplementary Figure 1

MALDI-TOF MS spectrum and mapping data of the modified tryptic peptide of apoA-I treated with 0.8 mM HcyT.

(A) The spectrum confirms the presence of *N*-homocysteinylation at a lysine residue. The precursor ion had an m/z value of 2056.06 and was fragmented using LIFT mode on an UltrafleXtreme MALDI-TOF mass spectrometer (Bruker). (B) The underlined sequence represents the detected amino acid sequence of the tryptic peptide confirmed by MALDI-TOF MS. The sequence coverage rate was 89%. The amino acid sequence is based on Uniprot entry for apoA-I protein. apoA-I, apolipoprotein A-I; HcyT, homocysteine-thiolactone; MALDI-TOF MS, matrix assisted laser desorption ionization time-of-flight mass spectrometry.

A

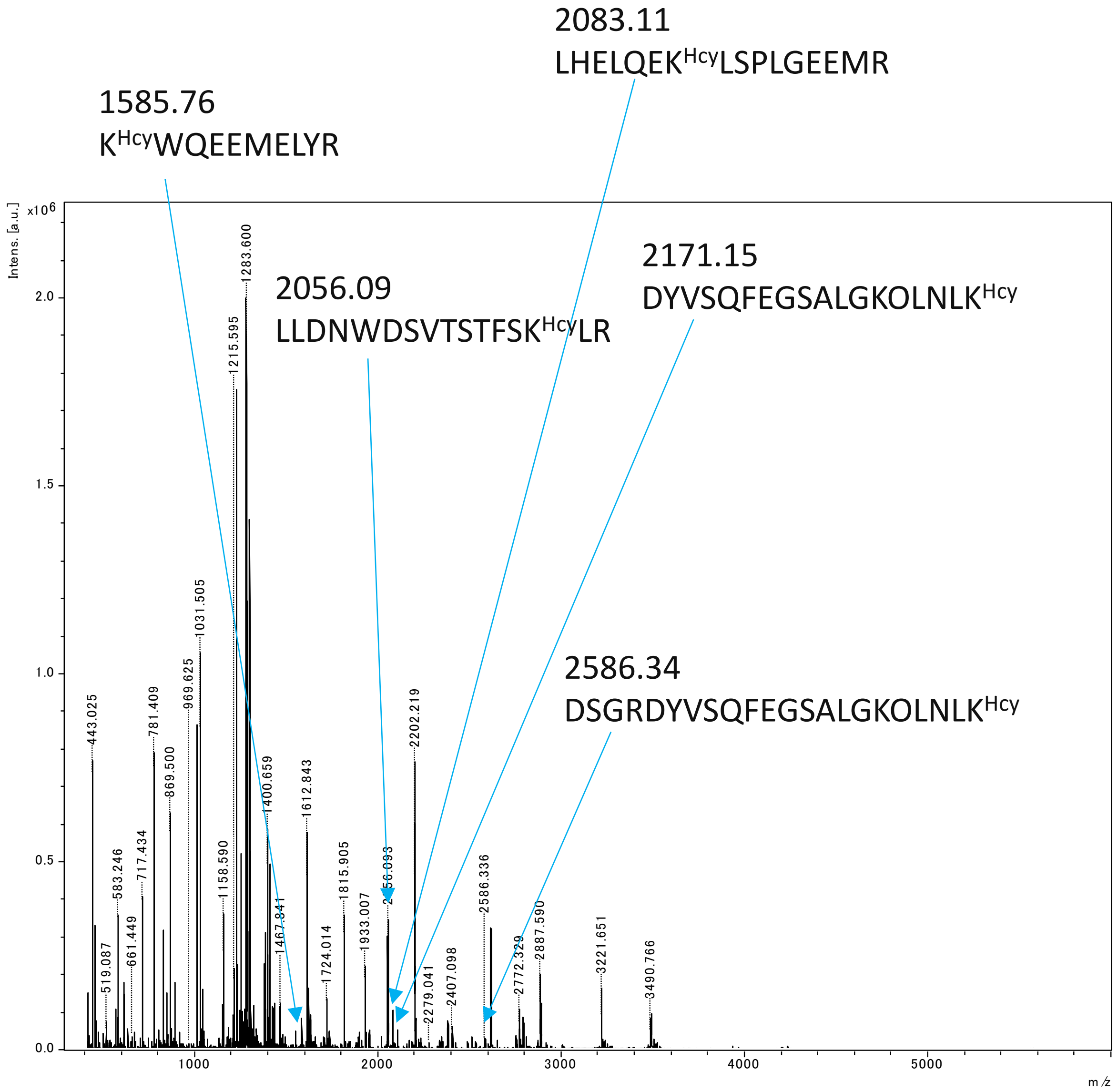

B

```
MKAAVLTLAV LFLTGSQARH FWQQDEPPQS PWDRVKDLAT  40
VYVDVLKDSG RDYVSQFEGS ALGKQLNLKL LDNWDSVTST  80
FSKLREQLGP VTQEFWDNLE KETEGLRQEM SKDLEEVKAK 120
VQPYLDDFQK KWQEEMELYR QKVEPLRAEL QEGARQKLHE 160
LQEKLSPLGE EMRDRARAHV DALRTHLAPY SDELRQRLAA 200
RLEALKENGG ARLAEYHAKA TEHLSTLSEK AKPALEDLRQ 240
GLLPVLESFK VSFLSALEEY TKKLNTQ 267
```

Supplementary Figure 2

MALDI-TOF MS spectrum and mapping data of the modified tryptic peptide of apoA-I treated with 4.0 mM HcyT.

(A) The spectrum confirms the presence of *N*-homocysteinylation at one or more lysine residues. The precursor ion had m/z values of 1585.76, 2056.09, 2083.11, 2171.15, and 2586.34 and were fragmented using LIFT mode on an UltrafleXtreme MALDI-TOF mass spectrometer (Bruker). (B) The underlined sequence represents the detected amino acid sequence of the tryptic peptide confirmed by MALDI-TOF MS. The sequence coverage rate was 83%. The amino acid sequence is based on Uniprot entry for apoA-I protein. apoA-I, apolipoprotein A-I; HcyT, homocysteine-thiolactone; MALDI-TOF MS, matrix assisted laser desorption ionization time-of-flight mass spectrometry.

A

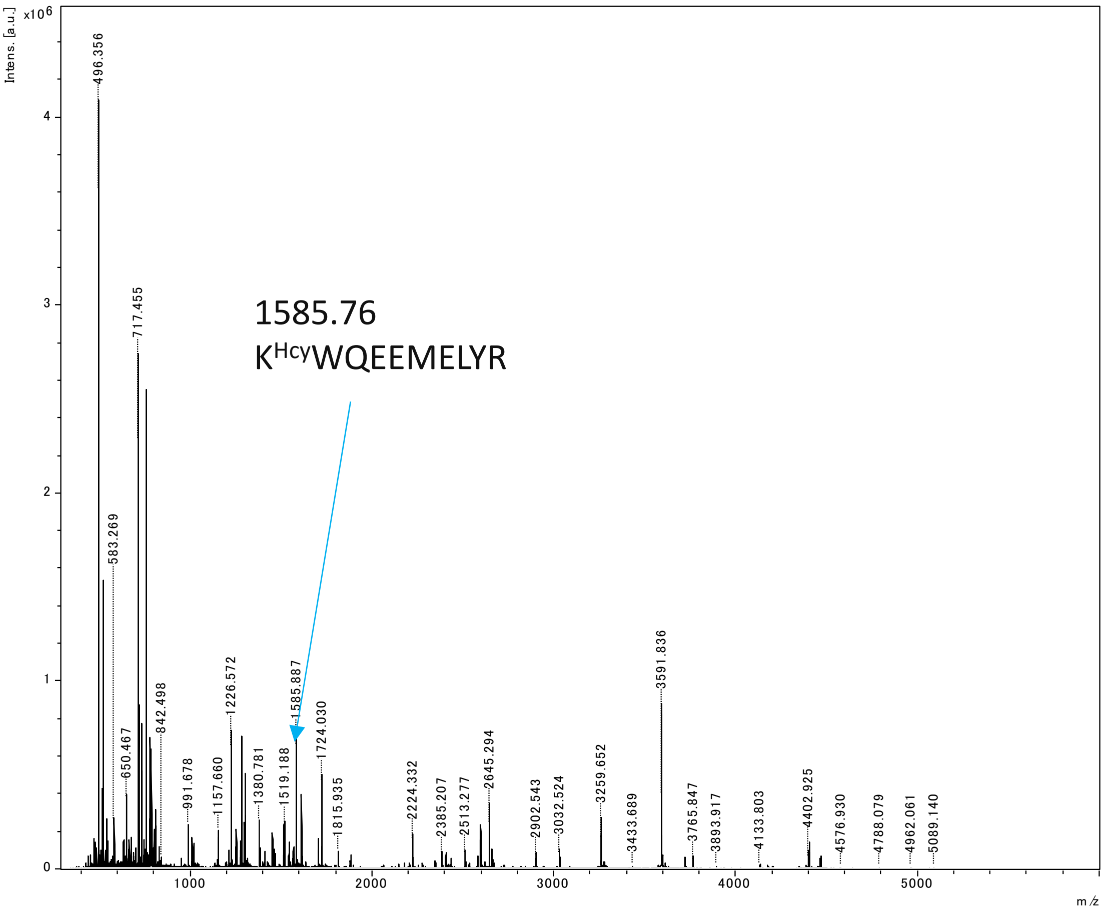

B

MKAAVLTLAV LFLTGSQARH FWQQDEPPQS PWDRVKDLAT 40
VYVDVLKDSG RDYVSQFEGS ALGKQLNLKL LDNWDSVTST 80
FSKLREQLGP VTQEFWDNLE KETEGLRQEM SKDLEEVKAK 120
VQPYLDDFQK KWQEEMELYR QKVEPLRAEL QEGARQKLHE 160
LQEKLSPLGE EMRDRARAHV DALRTHLAPY SDELRQRLAA 200
RLEALKENGG ARLAEYHAKA TEHLSTLSEK AKPALEDLRQ 240
GLLPVLESFK VSFLSALEEY TKKLNTQ 267

Supplementary Figure 3

MALDI-TOF MS spectrum and mapping data of the modified tryptic peptide of apoA-I contained within reconstituted HDL treated with 1.5 mM HcyT.

(A) The spectrum confirms the presence of *N*-homocysteinylation at a lysine residue. The precursor ion had an m/z value of 1585.76 and was fragmented using LIFT mode on an UltrafleXtreme MALDI-TOF mass spectrometer (Bruker). (B) The underlined sequence represents the detected amino acid sequence of the tryptic peptide confirmed by MALDI-TOF MS. The sequence coverage rate was 87%. The amino acid sequence is based on Uniprot entry for apoA-I protein. apoA-I, apolipoprotein A-I; HcyT, homocysteine-thiolactone; MALDI-TOF MS, matrix assisted laser desorption ionization time-of-flight mass spectrometry.

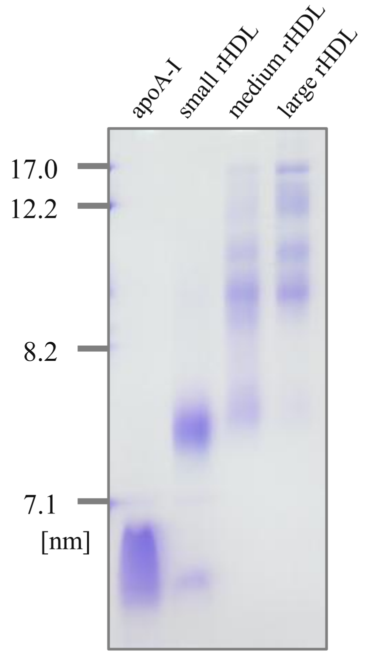

Supplementary Figure 4
Particle size of reconstituted HDL (rHDL).
Purified apoA-I and rHDL were analyzed by Native-PAGE using a 4-20% non-denaturing polyacrylamide gradient gel and staining with Coomassie Brilliant Blue (CBB) (4 µg protein/lane). Representative profiles from two independent experiments are shown. apoA-I, apolipoprotein A-I.

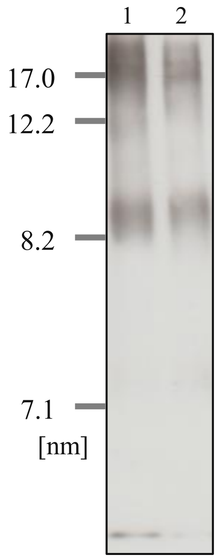

Lane 1: rHDL composed of untreated apoA-I

Lane 2: rHDL composed of Hcy-thiolactone treated apoA-I

Supplementary Figure 5

Particle size of reconstituted HDL (rHDL) isolated for Hcy-thiolactone treated apoA-I. Purified apoA-I was treated with 4.0 mM Hcy-thiolactone and rHDL was produced from untreated or treated apoA-I. Large rHDL with rePON1 were analyzed by Native-PAGE using a 7% non-denaturing polyacrylamide gel and western blotting of apoA-I (0.5 μg protein/lane). Representative profiles from three independent experiments are shown. apoA-I, apolipoprotein A-I.

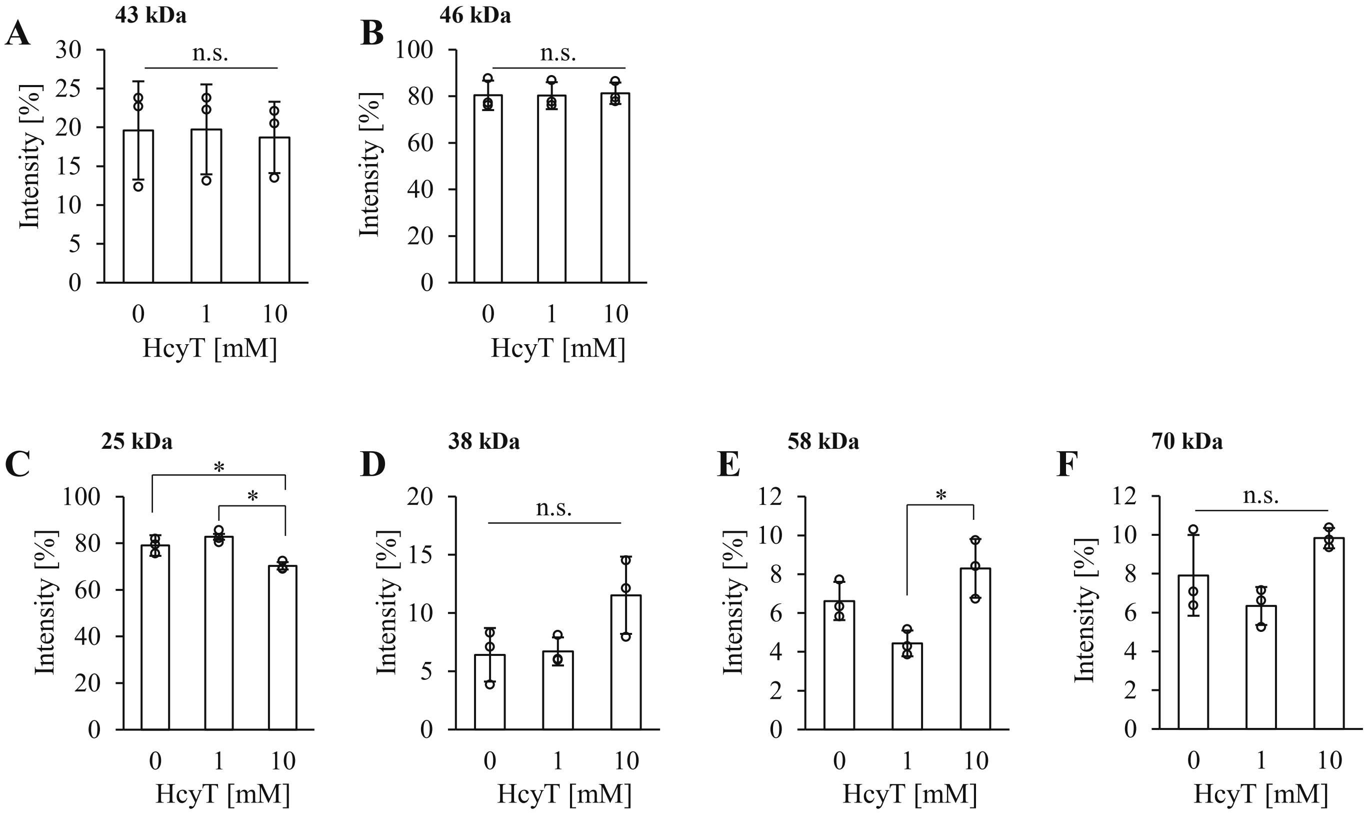

Supplementary Figure 6

Quantification result of PON1 and apoA-I protein assessment in uHDL samples.

Images of figure 3A and 3E were analyzed by densitometry on a CS Analyzer 4. A percentage of total intensity of PON1 bands are shown under reducing conditions at approximately 43(A), and 46 kDa(B). A percentage of total intensity of apoA-I bands are shown under reducing conditions at approximately 25(C), 38(D), 58(E), and 70 kDa(F). Data are presented as mean ± SD. *P < 0.05 determined by one-way analysis of variance with Tukey correction or Games-Howell correction. n.s., not significant; HcyT, homocysteine-thiolactone; uHDL, HDL fraction isolated by ultracentrifugation; PON1, paraoxonase 1; apoA-I, apolipoprotein A-I.

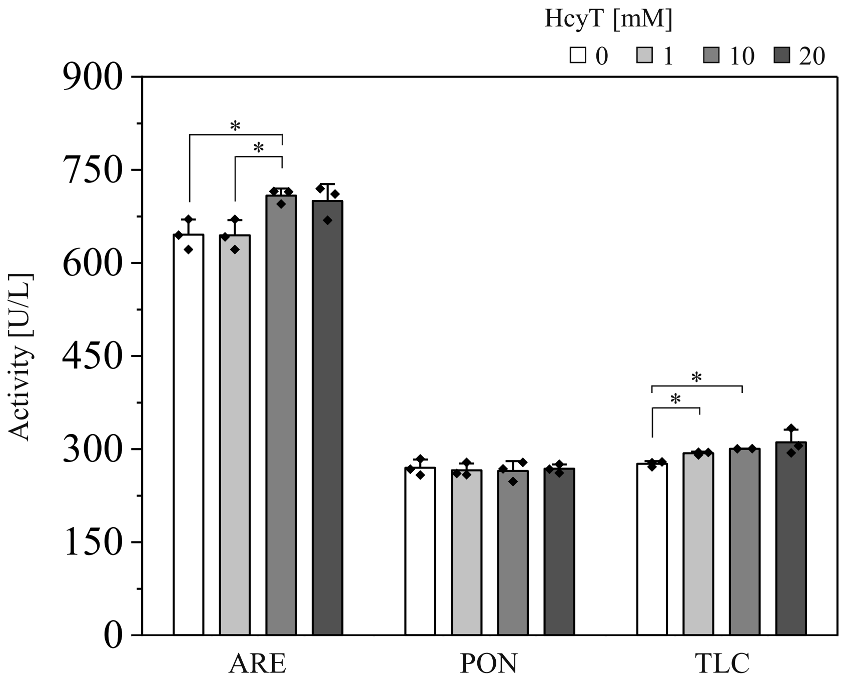

Supplementary Figure 7

PON1 activities in serum immediately after homocysteine-thiolactone addition.

PON1 activities were measured immediately after adding different concentrations of HcyT to serum: arylesterase (ARE) activity; paraoxonase (PON) activity; and thiolactonase (TLC) activity. Data are presented as mean ± SD. *$P < 0.05$ determined by one-way analysis of variance with Tukey correction or Games-Howell correction. HcyT, homocysteine-thiolactone.

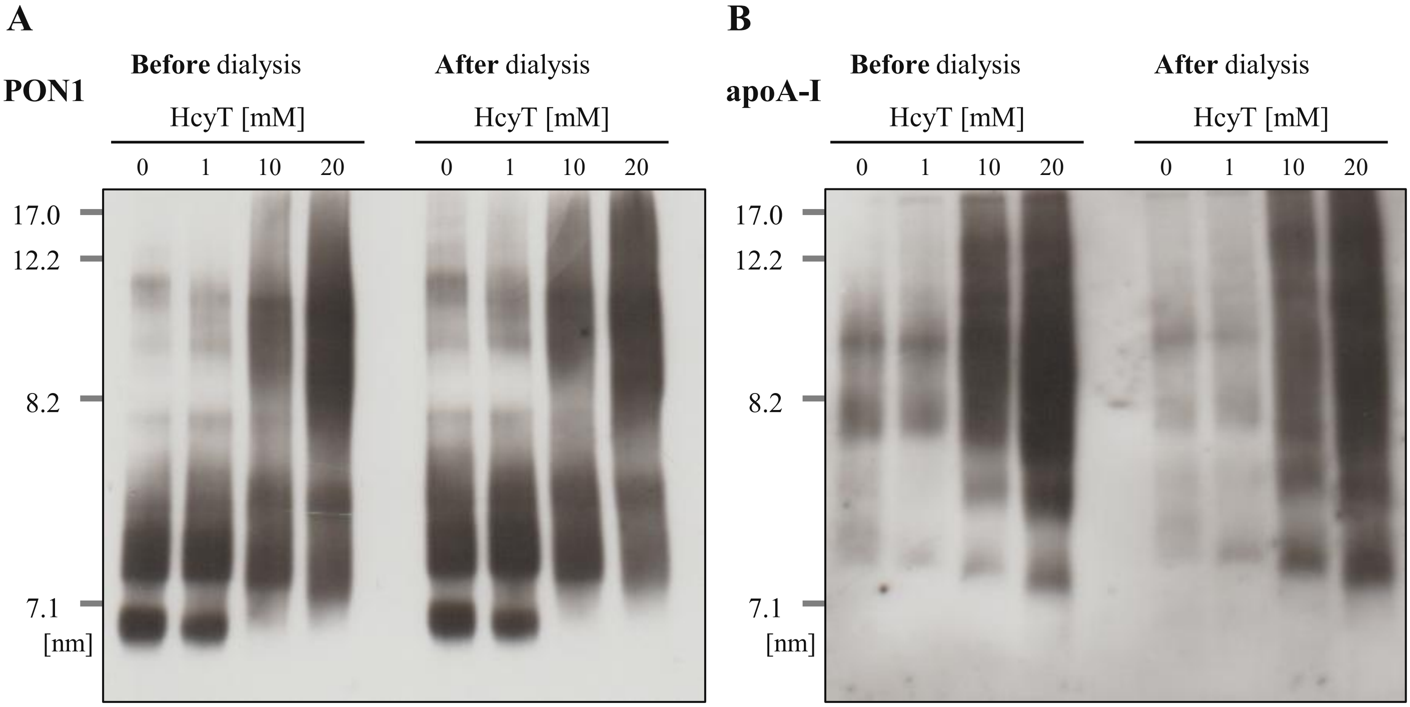

Supplementary Figure 8

Changes in PON1 distribution on HDL particles before and after Hcy-thiolactone removal.
Serum samples (45 mg HDL-C/dL) were incubated with 0, 1, 10, and 20 mM HcyT at 37°C for 24 h. Reversibility of the PON1 distribution change was assessed by removing Hcy-thiolactone via dialysis against the PON1 buffer. Representative western blots of PON1 (A) and apoA-I (B) by Native-PAGE (0.34 µg HDL protein/lane) are shown from three independent experiments. HcyT, homocysteine-thiolactone; PON1, paraoxonase 1; apoA-I, apolipoprotein A-I.

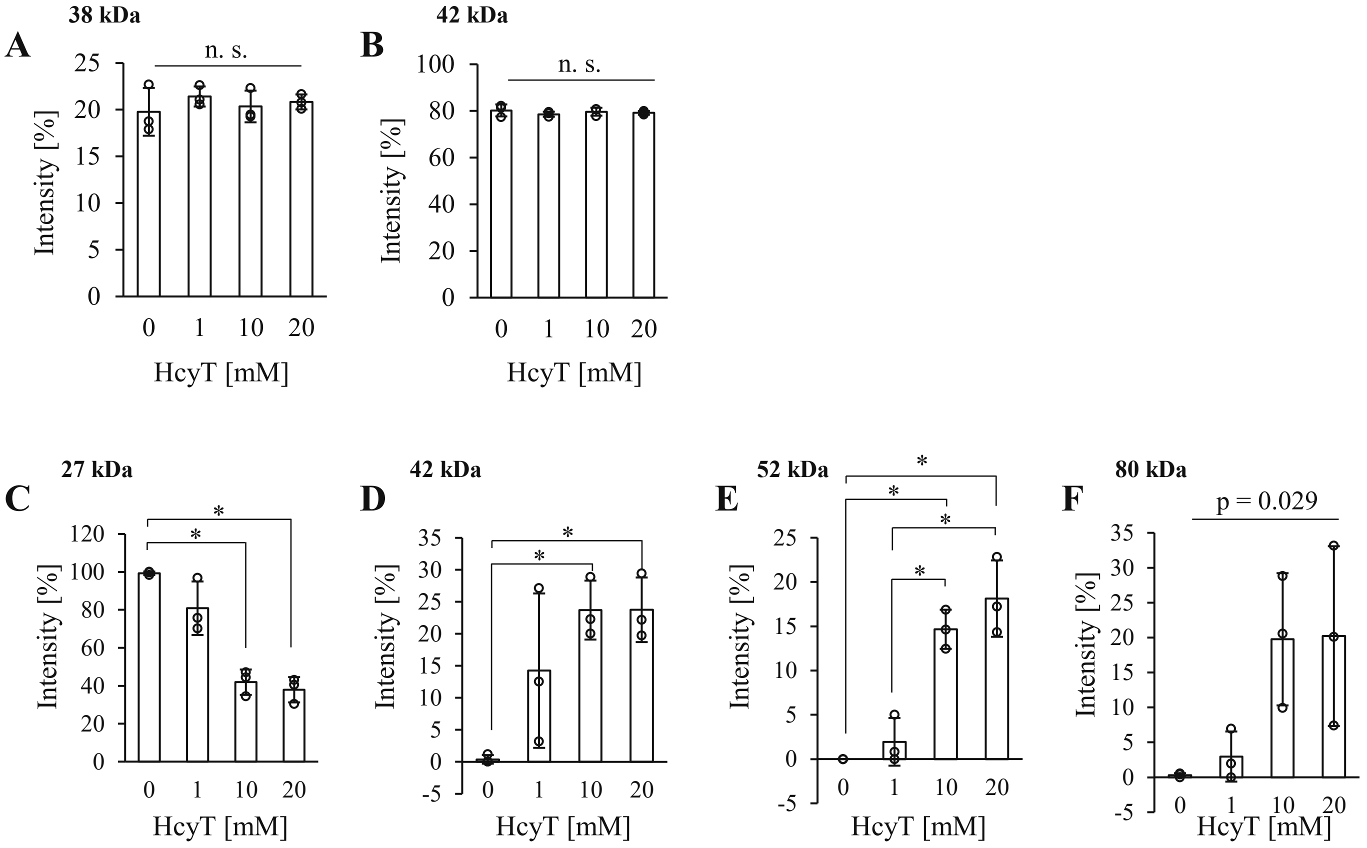

Supplementary Figure 9

Quantification result of PON1 and apoA-I protein assessment in serum samples.

Images of figure 5A and 5G were analyzed by densitometry on a CS Analyzer 4. A percentage of total intensity of PON1 are shown under reducing conditions at approximately 38(A), and 42 kDa(B). A percentage of total intensity of apoA-I are shown without reduction condition at approximately 27(C), 42(D), 52(E), and 80 kDa(F). Data are presented as mean ± SD. *P < 0.05 compared by one-way analysis of variance with Tukey correction or Games-Howell correction. n.s., not significant; HcyT, homocysteine-thiolactone; PON1, paraoxonase 1; apoA-I, apolipoprotein A-I.
