# Supplementary material for: Homocysteine thiolactone affects paraoxonase 1 activity via altered paraoxonase 1 distribution on high-density lipoprotein particles
Source: Biochem J. 2026 Jan 22;46(Pt 1):BSR20253768. doi: 10.1042/BSR20253768 (PMC12905485; doi:10.1042/BSR20253768)
Supplement: online supplementary table 1 [file bcj-46-1-BSR20253768-s002.docx]

**Supplementary Table 1**

**MS/MS fragment ions calculated and observed for the modified tryptic peptide (LLDNWDSVTSTFSK^Hcy^LR) encompassing Lys83 in apoA-I after treatment with 0.8 mM HcyT.**

The table lists the calculated MS/MS fragment ions (black) that correspond to the experimentally observed *y*-ions and *b*-ions (red). The *N*-homocysteinylation site is confirmed at Lys83 (K^Hcy^) in apoA-I following 0.8 mM HcyT treatment.

| # | *a* | *a** | *b* | *b** | Seq. | *y* | *y** | # |
| --- | --- | --- | --- | --- | --- | --- | --- | --- |
| 1 | 86.10 |  | 114.09 |  | L |  |  | 16 |
| 2 | 199.18 |  | 227.18 |  | L | 1942.93 | 1925.91 | 15 |
| 3 | 314.21 |  | 342.20 |  | D | 1829.85 | **1812.82** | 14 |
| 4 | 428.25 | 411.22 | **456.25** | 439.22 | N | **1714.82** | 1697.80 | 13 |
| 5 | 614.33 | 597.30 | 642.32 | 625.30 | W | **1600.78** | 1583.75 | 12 |
| 6 | 729.36 | 712.33 | 757.35 | 740.32 | D | **1414.70** | 1397.67 | 11 |
| 7 | 816.39 | 799.36 | 844.38 | 827.36 | S | **1299.67** | 1282.65 | 10 |
| 8 | 915.46 | 898.43 | 943.45 | 926.43 | V | **1212.64** | 1195.61 | 9 |
| 9 | 1016.50 | 999.48 | 1044.50 | 1027.47 | T | **1113.57** | 1096.55 | 8 |
| 10 | 1103.54 | 1086.51 | 1131.53 | 1114.51 | S | **1012.52** | 995.50 | 7 |
| 11 | 1204.58 | 1187.56 | 1232.58 | 1215.55 | T | **925.49** | 908.47 | 6 |
| 12 | 1351.65 | 1334.63 | 1379.65 | 1362.62 | F | **824.44** | 807.42 | 5 |
| 13 | 1438.68 | 1421.66 | 1466.68 | 1449.65 | S | **677.38** | **660.35** | 4 |
| 14 | 1740.83 | 1723.80 | 1768.82 | 1751.79 | K | 590.34 | 573.32 | 3 |
| 15 | 1853.91 | 1836.88 | 1881.91 | 1864.88 | L | **288.20** | 271.18 | 2 |
| 16 |  |  |  |  | R | **175.12** | 158.09 | 1 |

apoA-I, apolipoprotein A-I; HcyT, homocysteine thiolactone

**Supplementary Table 2**

**MS/MS fragment ions calculated and observed for the modified tryptic peptide (DSGRDYVSQFEGSALGKOLNLK^Hcy^) encompassing Lys69 in apoA-I after treatment with 4.0 mM HcyT.**

The table lists the calculated MS/MS fragment ions (black) that correspond to the experimentally observed *y*-ions and *b*-ions (red). The *N*-homocysteinylation site is confirmed at Lys69 (K^Hcy^) in apoA-I following 4.0 mM HcyT treatment.

| # | *a* | *a** | *b* | *b** | Seq. | *y* | *y** | # |
| --- | --- | --- | --- | --- | --- | --- | --- | --- |
| 1 | 88.04 |  | 116.03 |  | D |  |  | 22 |
| 2 | **175.07** |  | 203.07 |  | S | **2471.23** | 2454.21 | 21 |
| 3 | 232.09 |  | 260.09 |  | G | 2384.20 | 2367.18 | 20 |
| 4 | 388.19 | 371.17 | **416.19** | **399.16** | R | 2327.18 | 2310.15 | 19 |
| 5 | 503.22 | 486.19 | **531.22** | 514.19 | D | 2171.08 | 2154.05 | 18 |
| 6 | 666.28 | 649.26 | 694.28 | 677.25 | Y | 2056.05 | 2039.03 | 17 |
| 7 | 765.35 | 748.33 | 793.35 | 776.32 | V | 1892.99 | 1875.96 | 16 |
| 8 | 852.38 | 835.36 | 880.38 | 863.35 | S | 1793.92 | 1776.89 | 15 |
| 9 | 980.44 | 963.42 | 1008.44 | 991.41 | Q | 1706.89 | 1689.86 | 14 |
| 10 | 1127.51 | 1110.49 | 1155.51 | 1138.48 | F | 1578.83 | 1561.8 | 13 |
| 11 | 1256.55 | 1239.53 | **1284.55** | 1267.52 | E | 1431.76 | 1414.74 | 12 |
| 12 | 1313.58 | 1296.55 | 1341.57 | 1324.54 | G | 1302.72 | 1285.69 | 11 |
| 13 | 1400.61 | 1383.58 | 1428.60 | 1411.58 | S | 1245.70 | 1228.67 | 10 |
| 14 | 1471.64 | 1454.62 | 1499.64 | 1482.61 | A | 1158.67 | 1141.64 | 9 |
| 15 | 1584.73 | 1567.70 | 1612.72 | 1595.70 | L | 1087.63 | 1070.60 | 8 |
| 16 | 1641.75 | 1624.72 | 1669.75 | 1652.72 | G | 974.55 | 957.52 | 7 |
| 17 | 1769.85 | 1752.82 | 1797.84 | 1780.81 | K | 917.52 | 900.50 | 6 |
| 18 | 1897.90 | 1880.88 | 1925.90 | 1908.87 | Q | 789.43 | 772.40 | 5 |
| 19 | 2010.99 | 1993.96 | 2038.98 | 2021.96 | L | 661.37 | 644.34 | 4 |
| 20 | 2125.03 | 2108.00 | 2153.03 | 2136.00 | N | 548.29 | **531.26** | 3 |
| 21 | 2238.11 | 2221.09 | 2266.11 | 2249.08 | L | 434.24 | 417.22 | 2 |
| 22 |  |  |  |  | K | 321.16 | 304.13 | 1 |

apoA-I, apolipoprotein A-I; HcyT, homocysteine thiolactone

**Supplementary Table 3**

**MS/MS fragment ions calculated and observed for the modified tryptic peptide (DYVSQFEGSALGKOLNLK^Hcy^) encompassing Lys69 in apoA-I after treatment with 4.0 mM HcyT.**

The table lists the calculated MS/MS fragment ions (black) that correspond to the experimentally observed *y*-ions and *b*-ions (red). The *N*-homocysteinylation site is confirmed at Lys69 (K^Hcy^) in apoA-I following 4.0 mM HcyT treatment.

| # | *a* | *a** | *b* | *b** | Seq. | *y* | *y** | # |
| --- | --- | --- | --- | --- | --- | --- | --- | --- |
| 1 | 88.04 |  | 116.03 |  | D |  |  | 18 |
| 2 | 251.10 |  | 279.10 |  | Y | 2056.05 | 2039.03 | 17 |
| 3 | 350.17 |  | 378.17 |  | V | 1892.99 | 1875.96 | 16 |
| 4 | 437.20 |  | 465.20 |  | S | 1793.92 | 1776.89 | 15 |
| 5 | 565.26 | 548.24 | 593.26 | 576.23 | Q | 1706.89 | 1689.86 | 14 |
| 6 | 712.33 | 695.30 | 740.32 | 723.30 | F | 1578.83 | 1561.80 | 13 |
| 7 | 841.37 | 824.35 | 869.37 | 852.34 | E | 1431.76 | 1414.74 | 12 |
| 8 | 898.39 | 881.37 | 926.39 | 909.36 | G | **1302.72** | 1285.69 | 11 |
| 9 | 985.43 | 968.40 | 1013.42 | 996.39 | S | 1245.70 | 1228.67 | 10 |
| 10 | 1056.46 | 1039.44 | 1084.46 | 1067.43 | A | **1158.67** | 1141.64 | 9 |
| 11 | 1169.55 | 1152.52 | 1197.54 | 1180.52 | L | **1087.63** | 1070.60 | 8 |
| 12 | 1226.57 | 1209.54 | 1254.56 | 1237.54 | G | **974.55** | 957.52 | 7 |
| 13 | 1354.66 | 1337.64 | 1382.66 | 1365.63 | K | **917.52** | 900.50 | 6 |
| 14 | 1482.72 | 1465.70 | 1510.72 | 1493.69 | Q | 789.43 | 772.40 | 5 |
| 15 | 1595.81 | 1578.78 | 1623.80 | 1606.77 | L | 661.37 | 644.34 | 4 |
| 16 | 1709.85 | 1692.82 | 1737.84 | 1720.82 | N | 548.29 | 531.26 | 3 |
| 17 | 1822.93 | 1805.91 | 1850.93 | 1833.90 | L | 434.24 | 417.22 | 2 |
| 18 |  |  |  |  | K | 321.16 | 304.13 | 1 |

apoA-I, apolipoprotein A-I; HcyT, homocysteine thiolactone

**Supplementary Table 4**

**MS/MS fragment ions calculated and observed for the modified tryptic peptide (LLDNWDSVTSTFSK^Hcy^LR) encompassing Lys83 in apoA-I after treatment with 4.0 mM HcyT.**

The table lists the calculated MS/MS fragment ions (black) that correspond to the experimentally observed *y*-ions and *b*-ions (red). The *N*-homocysteinylation site is confirmed at Lys83 (K^Hcy^) in apoA-I following 4.0 mM HcyT treatment.

| # | *a* | *a** | *b* | *b** | Seq. | *y* | *y** | # |
| --- | --- | --- | --- | --- | --- | --- | --- | --- |
| 1 | 86.10 |  | 114.09 |  | L |  |  | 16 |
| 2 | 199.18 |  | 227.18 |  | L | 1942.93 | 1925.91 | 15 |
| 3 | 314.21 |  | 342.20 |  | D | 1829.85 | **1812.82** | 14 |
| 4 | 428.25 | 411.22 | **456.25** | 439.22 | N | **1714.82** | 1697.80 | 13 |
| 5 | 614.33 | 597.30 | 642.32 | 625.30 | W | **1600.78** | 1583.75 | 12 |
| 6 | 729.36 | 712.33 | 757.35 | 740.32 | D | **1414.70** | 1397.67 | 11 |
| 7 | 816.39 | 799.36 | 844.38 | 827.36 | S | **1299.67** | 1282.65 | 10 |
| 8 | 915.46 | 898.43 | 943.45 | 926.43 | V | **1212.64** | 1195.61 | 9 |
| 9 | 1016.50 | 999.48 | 1044.50 | 1027.47 | T | **1113.57** | 1096.55 | 8 |
| 10 | 1103.54 | 1086.51 | 1131.53 | 1114.51 | S | **1012.52** | 995.50 | 7 |
| 11 | 1204.58 | 1187.56 | 1232.58 | 1215.55 | T | **925.49** | 908.47 | 6 |
| 12 | 1351.65 | 1334.63 | 1379.65 | 1362.62 | F | **824.44** | 807.42 | 5 |
| 13 | 1438.68 | 1421.66 | 1466.68 | 1449.65 | S | **677.38** | **660.35** | 4 |
| 14 | 1740.83 | 1723.80 | 1768.82 | 1751.79 | K | 590.34 | 573.32 | 3 |
| 15 | 1853.91 | 1836.88 | 1881.91 | 1864.88 | L | **288.20** | 271.18 | 2 |
| 16 |  |  |  |  | R | **175.12** | 158.09 | 1 |

apoA-I, apolipoprotein A-I; HcyT, homocysteine thiolactone

**Supplementary Table 5**

**MS/MS fragment ions calculated and observed for the modified tryptic peptide (K^Hcy^WQEEMELYR) encompassing Lys131 in apoA-I after treatment with 4.0 mM HcyT.**

The table lists the calculated MS/MS fragment ions (black) that correspond to the experimentally observed *y*-ions and *b*-ions (red). The *N*-homocysteinylation site is confirmed at Lys131 (K^Hcy^) in apoA-I following 4.0 mM HcyT treatment.

| # | *a* | *a** | *b* | *b** | Seq. | *y* | *y** | # |
| --- | --- | --- | --- | --- | --- | --- | --- | --- |
| 1 | 275.15 | 258.13 | 303.15 | 286.12 | K |  |  | 10 |
| 2 | 461.23 | 444.21 | **489.23** | 472.20 | W | 1283.57 | 1266.55 | 9 |
| 3 | 589.29 | 572.26 | **617.29** | 600.26 | Q | 1097.49 | 1080.47 | 8 |
| 4 | 718.33 | 701.31 | 746.33 | 729.30 | E | 969.43 | 952.41 | 7 |
| 5 | 847.38 | 830.35 | 875.37 | 858.35 | E | **840.39** | 823.37 | 6 |
| 6 | 978.42 | 961.39 | 1006.41 | 989.39 | M | **711.35** | 694.32 | 5 |
| 7 | 1107.46 | 1090.43 | 1135.45 | 1118.43 | E | 580.31 | 563.28 | 4 |
| 8 | 1220.54 | 1203.52 | 1248.54 | 1231.51 | L | **451.27** | 434.24 | 3 |
| 9 | 1383.61 | 1366.58 | **1411.60** | 1394.58 | Y | **338.18** | 321.16 | 2 |
| 10 |  |  |  |  | R | **175.12** | 158.09 | 1 |

apoA-I, apolipoprotein A-I; HcyT, homocysteine thiolactone

**Supplementary Table 6**

**MS/MS fragment ions calculated and observed for the modified tryptic peptide (LHELQEK^Hcy^LSPLGEEMR) encompassing Lys164 in apoA-1 after treatment with 4.0 mM HcyT.**

The table lists the calculated MS/MS fragment ions (black) that correspond to the experimentally observed *y*-ions and *b*-ions (red). The *N*-homocysteinylation site is confirmed at Lys164 (K^Hcy^) in apoA-I following 4.0 mM HcyT treatment.

| # | *a* | *a** | *b* | *b** | Seq. | *y* | *y** | # |
| --- | --- | --- | --- | --- | --- | --- | --- | --- |
| 1 | 86.10 |  | 114.09 |  | L |  |  | 16 |
| 2 | 223.16 |  | **251.15** |  | H | 1969.95 | 1952.92 | 15 |
| 3 | 352.20 |  | **380.19** |  | E | 1832.89 | 1815.86 | 14 |
| 4 | 465.28 |  | **493.28** |  | L | **1703.85** | 1686.82 | 13 |
| 5 | 593.34 | 576.31 | **621.34** | 604.31 | Q | 1590.76 | 1573.73 | 12 |
| 6 | 722.38 | 705.36 | **750.38** | 733.35 | E | 1462.70 | 1445.68 | 11 |
| 7 | 1024.52 | 1007.50 | **1052.52** | 1035.49 | K | **1333.66** | 1316.63 | 10 |
| 8 | 1137.61 | 1120.58 | **1165.60** | 1148.58 | L | **1031.52** | 1014.49 | 9 |
| 9 | 1224.64 | 1207.61 | 1252.64 | 1235.61 | S | **918.43** | 901.41 | 8 |
| 10 | 1321.69 | 1304.67 | 1349.69 | 1332.66 | P | **831.40** | 814.38 | 7 |
| 11 | 1434.78 | 1417.75 | 1462.77 | 1445.75 | L | 734.35 | 717.32 | 6 |
| 12 | 1491.80 | 1474.77 | 1519.79 | 1502.77 | G | **621.27** | 604.24 | 5 |
| 13 | 1620.84 | 1603.81 | 1648.84 | 1631.81 | E | 564.24 | 547.22 | 4 |
| 14 | 1749.88 | 1732.86 | 1777.88 | 1760.85 | E | 435.20 | 418.18 | 3 |
| 15 | 1880.92 | 1863.90 | 1908.92 | 1891.89 | M | 306.16 | 289.13 | 2 |
| 16 |  |  |  |  | R | 175.12 | 158.09 | 1 |

apoA-I, apolipoprotein A-I; HcyT, homocysteine thiolactone

**Supplementary Table 7**

**MS/MS fragment ions calculated and observed for the modified tryptic peptide (K^Hcy^WQEEMELYR) encompassing Lys131 in apoA-I contained within reconstituted HDL after treatment with 1.5 mM HcyT.**

The table lists the calculated MS/MS fragment ions (black) that correspond to the experimentally observed *y*-ions and *b*-ions (red). The *N*-homocysteinylation site is confirmed at Lys131 (K^Hcy^) in apoA-I contained within reconstituted HDL following 1.5 mM HcyT treatment.

| # | *a* | *a** | *b* | *b** | Seq. | *y* | *y** | # |
| --- | --- | --- | --- | --- | --- | --- | --- | --- |
| 1 | 275.15 | 258.13 | 303.15 | 286.12 | K |  |  | 10 |
| 2 | 461.23 | 444.21 | **489.23** | 472.20 | W | 1283.57 | 1266.55 | 9 |
| 3 | 589.29 | 572.26 | **617.29** | 600.26 | Q | 1097.49 | 1080.47 | 8 |
| 4 | 718.33 | 701.31 | 746.33 | 729.30 | E | 969.43 | 952.41 | 7 |
| 5 | 847.38 | 830.35 | 875.37 | 858.35 | E | **840.39** | 823.37 | 6 |
| 6 | 978.42 | 961.39 | 1006.41 | 989.39 | M | **711.35** | 694.32 | 5 |
| 7 | 1107.46 | 1090.43 | 1135.45 | 1118.43 | E | 580.31 | 563.28 | 4 |
| 8 | 1220.54 | 1203.52 | 1248.54 | 1231.51 | L | **451.27** | 434.24 | 3 |
| 9 | 1383.61 | 1366.58 | **1411.60** | 1394.58 | Y | **338.18** | 321.16 | 2 |
| 10 |  |  |  |  | R | **175.12** | 158.09 | 1 |

apoA-I, apolipoprotein A-I; HcyT, homocysteine thiolactone

**Supplementary Table 8
Composition of reconstituted HDL (rHDL)**

|  | TP [µM] | PL [mM] | Chol [mM] |
| --- | --- | --- | --- |
| small rHDL | 0.142 | 0.368 | 0.043 |
| medium rHDL | 0.142 | 1.134 | 0.089 |
| large rHDL | 0.142 | 1.447 | 0.187 |

TP, total protein; PL, phospholipid; Chol, cholesterol

**Supplementary Table 9
PON1 activities in reconstituted HDL (rHDL)**

|  | ARE^a^ | PON^a^ | TLC^a^ |
| --- | --- | --- | --- |
| purified apoA-I | < 4.1 | < 1.5 | < 2.1 |
| small rHDL | < 4.1 | < 1.5 | < 2.1 |
| medium rHDL | < 4.1 | < 1.5 | < 2.1 |
| large rHDL | < 4.1 | < 1.5 | < 2.1 |

ARE, arylesterase activity; PON, paraoxonase activity; TLC, thiolactonase activity; apoA-I, apolipoprotein A-I

^a^PON1 activity in purified apoA-I or reconstituted HDL (0.1 mg protein/mL) was measured.
